# Regional Differences in Human Biliary Tissues and Corresponding *In Vitro*–Derived Organoids

**DOI:** 10.1002/hep.31252

**Published:** 2021-02-06

**Authors:** Casey A. Rimland, Samantha G. Tilson, Carola M. Morell, Rute A. Tomaz, Wei‐Yu Lu, Simone E. Adams, Nikitas Georgakopoulos, Francisco Otaizo‐Carrasquero, Timothy G. Myers, John R. Ferdinand, Richard L. Gieseck, Fotios Sampaziotis, Olivia C. Tysoe, Alexander Ross, Judith M. Kraiczy, Brandon Wesley, Daniele Muraro, Matthias Zilbauer, Gabriel C. Oniscu, Nicholas R.F. Hannan, Stuart J. Forbes, Kourosh Saeb‐Parsy, Thomas A. Wynn, Ludovic Vallier

**Affiliations:** ^1^ Wellcome–Medical Research Council Cambridge Stem Cell Institute University of Cambridge Cambridge United Kingdom; ^2^ Immunopathogenesis Section Laboratory of Parasitic Diseases NIAID NIH Bethesda MD; ^3^ Department of Surgery University of Cambridge and National Institute for Health Research Cambridge Biomedical Research Centre Cambridge United Kingdom; ^4^ Medical Scientist Training Program School of Medicine University of North Carolina at Chapel Hill Chapel Hill NC; ^5^ Welcome Trust Sanger Institute Hinxton United Kingdom; ^6^ Liver Diseases Branch NIDDK NIH Bethesda MD; ^7^ MRC Centre for Regenerative Medicine University of Edinburgh Edinburgh United Kingdom; ^8^ Centre for Liver and Gastrointestinal Research Institute of Immunology and Immunotherapy The University of Birmingham Birmingham United Kingdom; ^9^ Department of Biological Sciences North Carolina State University Raleigh NC; ^10^ Genomic Technologies Section Research Technologies Branch NIAID NIH Bethesda MD; ^11^ Department of Medicine University of Cambridge Cambridge United Kingdom; ^12^ Department of Paediatrics University of Cambridge Cambridge United Kingdom; ^13^ Edinburgh Transplant Centre Royal Infirmary of Edinburgh University of Edinburgh Edinburgh United Kingdom; ^14^ Division of Cancer and Stem Cells School of Medicine Centre for Biomolecular Sciences University of Nottingham Nottingham United Kingdom; ^15^ National Institute for Health Research Nottingham Digestive Diseases Biomedical Research Unit Nottingham University Hospitals NHS Trust and University of Nottingham Nottingham United Kingdom

## Abstract

**Background and Aims:**

Organoids provide a powerful system to study epithelia *in vitro*. Recently, this approach was applied successfully to the biliary tree, a series of ductular tissues responsible for the drainage of bile and pancreatic secretions. More precisely, organoids have been derived from ductal tissue located outside (extrahepatic bile ducts; EHBDs) or inside the liver (intrahepatic bile ducts; IHBDs). These organoids share many characteristics, including expression of cholangiocyte markers such as keratin (KRT) 19. However, the relationship between these organoids and their tissues of origin, and to each other, is largely unknown.

**Approach and Results:**

Organoids were derived from human gallbladder, common bile duct, pancreatic duct, and IHBDs using culture conditions promoting WNT signaling. The resulting IHBD and EHBD organoids expressed stem/progenitor markers leucine‐rich repeat–containing G‐protein‐coupled receptor 5/prominin 1 and ductal markers KRT19/KRT7. However, RNA sequencing revealed that organoids conserve only a limited number of regional‐specific markers corresponding to their location of origin. Of particular interest, down‐regulation of biliary markers and up‐regulation of cell‐cycle genes were observed in organoids. IHBD and EHBD organoids diverged in their response to WNT signaling, and only IHBDs were able to express a low level of hepatocyte markers under differentiation conditions.

**Conclusions:**

Taken together, our results demonstrate that differences exist not only between extrahepatic biliary organoids and their tissue of origin, but also between IHBD and EHBD organoids. This information may help to understand the tissue specificity of cholangiopathies and also to identify targets for therapeutic development.

**Abbreviations**
3D: three‐dimensional
ADV/DMEM‐F12: advanced DMEM‐F12
ALB: albumin
ANOVAs: analyses of variance
CBD: common bile duct
CDX2: caudal‐related homeobox 2
CFTR: cystic fibrosis transmembrane conductance regulator
CYP3A4: cytochrome P450 family 3 subfamily A member 4
DKK‐1: dickkopf WNT signaling pathway inhibitor 1
DMEM: Dulbecco’s modified Eagle’s medium
ECOs: extrahepatic cholangiocyte organoids
EGF: epidermal growth factor
EHBDs: extrahepatic bile ducts
EPCAM: epithelial cell adhesion molecule
FSK: forskolin
GBD: gallbladder
GGT: gamma‐glutamyltransferase
GFR: growth factor reduced
GO: Gene Ontology
GSK3β: glycogen synthase kinase 3 beta
HNF1B: hepatocyte nuclear factor 1 beta
HNF4A: hepatocyte nuclear factor 4 alpha
HOXB2: homeobox 2
IF: immunofluorescence
IHBDs: intrahepatic bile ducts
KRT: keratin
LGR5: leucine‐rich repeat–containing G‐protein‐coupled receptor 5
MUC1: mucin 1, cell‐surface associated
PancD: pancreatic duct
PCA: principal component analysis
PROM1: prominin 1
RNA‐Seq: RNA sequencing
RSPO‐1: R‐spondin 1
SOX9: SRY‐box transcription factor 9
SOX17: SRY‐box transcription factor 17
SPP1: secreted phosphoprotein 1
TBX3: T‐box 3
TTR: transthyretin
UGT1A6: UDP glucuronosyltransferase family 1 member A6 [UGT1A6]

The biliary tree is a series of ductular tissues lined by epithelial cells called cholangiocytes. It is responsible for the drainage, storage, and concentration of bile produced by the liver and pancreatic secretions from the pancreas.^(^ ^1^ ^)^ The biliary tree is broadly divided into two compartments: intra‐ and extrahepatic.^(^ ^1^, ^2^ ^)^ The intrahepatic compartment is further divided based on duct size, whereas the extrahepatic compartment is divided by anatomical region, including the common hepatic duct, gallbladder (GBD), cystic duct, common bile duct (CBD), and pancreatic duct (PancD).^(^ ^2^ ^)^ Embryologically, these compartments arise from different precursor cells.^(^ ^3^ ^)^ Intrahepatic cholangiocytes arise from bipotent hepatoblasts, whereas extrahepatic cholangiocytes share an embryologic origin with the ventral pancreas.^(^ ^3^ ^)^

Research on cholangiocytes has historically focused on the intrahepatic compartment.^(^ ^4^, ^5^ ^)^ Analysis of intrahepatic bile ducts has demonstrated the existence of two populations of cholangiocytes: small and large, which respectively line the smallest intrahepatic ductules and the larger intrahepatic ducts.^(^ ^4^, ^5^ ^)^ It has been shown that large and small cholangiocytes display different transcriptional profiles, proliferative capacity, and biological function.^(^ ^4^, ^5^ ^)^ On the other hand, little is known about the diversity that may exist between individual anatomical regions of the extrahepatic compartment. This understanding is critical given that human cholangiopathies, such as biliary atresia, primary sclerosing cholangitis, and nonanastomotic biliary strictures, show regionalization and preferentially target extrahepatic bile ducts (EHBDs) early in disease.^(^ ^6^, ^7^, ^8^ ^)^

Additionally, it has been demonstrated that *in vitro* three‐dimensional (3D) culture of human biliary organoids is possible from both intra‐ and extrahepatic tissues.^(^ ^9^, ^10^, ^11^, ^12^ ^)^ However, it remains unclear whether the extrahepatic cultures contain stem/progenitor populations. Huch et al. isolated human intrahepatic bile ducts (IHBDs) in conditions promoting canonical WNT signaling. These resulting IHBD organoids were capable of long‐term expansion and bipotential differentiation toward either hepatocyte or biliary fates.^(^ ^9^ ^)^ This work suggested the presence of an intrahepatic stem/progenitor population. Using a similar WNT‐based system, Lugli et al. established both mouse and human GBD organoids.^(^ ^11^ ^)^ However, differentiation capacity was only assessed for the murine organoids and showed incomplete differentiation toward a hepatocyte fate with no *in vivo* differentiation potential.^(^ ^11^ ^)^ Furthermore, our group recently demonstrated that human cholangiocyte organoids from CBD and GBD, grown in the absence of canonical WNT, were capable of long‐term expansion, yet lacked characteristic markers of adult stem cells, such as leucine‐rich repeat–containing G‐protein‐coupled receptor 5 (LGR5) and prominin 1 (PROM1).^(^ ^12^ ^)^ The differentiation capacity of these organoids beyond a biliary fate was not assessed *in vitro*; however, these cells never generated hepatocytes after transplantation in mouse models.^(^ ^12^, ^13^ ^)^ Together, these reports suggest that both intra‐ and extrahepatic human cholangiocytes are capable of forming 3D‐organoid cultures, which, under certain conditions, display adult stem/progenitor phenotypes. However, it is unclear whether EHBD organoids are capable of bipotential differentiation like IHBD organoids. Additionally, how EHBD organoids from different anatomical regions of the biliary tree compare to each other, their tissue of origin, and IHBD organoids is also unknown. Here, we report the derivation and characterization of human biliary organoids from three regions of the human extrahepatic biliary tree: CBD, GBD, and PancD. We demonstrate that these EHBD organoids, when grown in conditions promoting canonical WNT signaling, express markers of adult stem cells, such as LGR5 and PROM1. Furthermore, we demonstrate that primary extrahepatic tissues display unique transcriptional signatures depending on anatomical origin. We also show that EHBD organoids maintain a limited number of these tissue‐specific differences. Last, we compare our EHBD to IHBD organoids and show that only IHBD organoids have the capacity to express hepatocyte‐specific markers when subjected to differentiation conditions.

## Materials and Methods

### Human Tissue Material

Human GBD, CBD, PancD, and liver samples were obtained from organ donors at either Cambridge University Hospitals or the Royal Infirmary of Edinburgh (REC reference: 16/WM/0093, 15/EE/0152). CBD, PancD, and IHBD organoid cell lines were derived from either liver or pancreas donors deemed unsuitable for transplant. GBD organoids were derived from livers deemed unsuitable or suitable for transplant, given that the GBD is not routinely retransplanted. Generally, the liver and pancreas were considered unsuitable for transplant because of excessively long ischemia time or evidence of disease (i.e., fatty liver or pancreatic calcifications). Informed consent for the use of tissues for research purposes was obtained from each donor’s next of kin, and protocols were reviewed and approved by the ethics committees at both hospitals and consent recorded according to the National Health Service Blood and Transplant consent regulation (FRM 1538). Tissue samples were stored at 4°C in University of Wisconsin Solution. Donor demographics are listed in Supporting Table S1.

### Extrahepatic Primary Epithelium Isolation and Organoid Culture

**Fig. 1:**
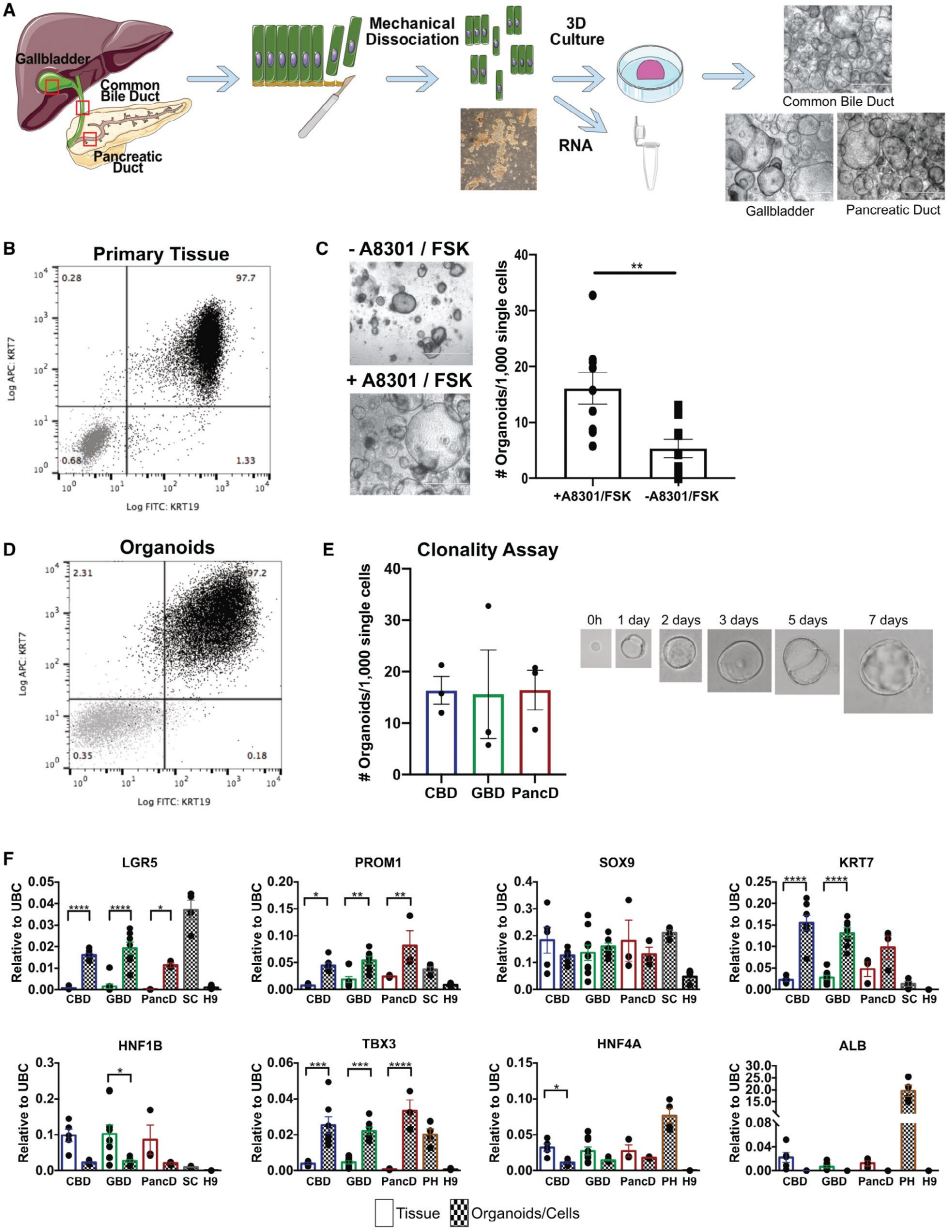
Derivation of extrahepatic organoids expressing biliary and stem/progenitor markers**.** (A) Diagram depicting the mechanical isolation used to obtain biliary epithelial cells, image showing the cells recovered, and representative images of organoids isolated from CBD, GBD, or PancD. Scale bars, 1,000 μm. (B) Representative flow cytometry analysis of mechanically dissociated epithelium from human GBD showing percentage of cells expressing KRT7 and KRT19 (black). Negative control: secondary antibody only (gray). (C) Representative image of deteroriated GBD organoid cultures derived without A 83‐01 and FSK that were rescued after 48 hours of treatment with A 83‐01 and FSK (scale bars, 1,000 μm). Graph showing clonality of EHBD organoids grown with or without A 83‐01 and FSK (mean and SEM; n = 9 independent experiments, n = 3 donors; unpaired *t* test). (D) Representative flow cytometry analysis of CBD organoids showing percentage of cells expressing KRT7 and KRT19 (black). Negative control: secondary antibody only (gray). (E) Clonality assay showing the number of organoids formed per 1,000 single cells for PancD, CBD, and GBD organoids (mean and SEM; n = 3 independent experiments, n = 1 donor line per EHBD region; one‐way ANOVA) and representative images of a single cell forming an organoid over 7 days. (F) qPCR showing expression of biliary and adult stem cell markers in EHBD organoids or tissue samples from CBD, GBD, and PancD. Organoids derived from human sigmoid colon (SC; n = 4), primary hepatocytes (PH; n = 4), and H9 embryonic stem cells (H9; n = 3) were used as controls. Gene expression is normalized to UBC and plotted as mean and SEM. CBD tissue (n = 5), CBD organoids at P5 (n = 7), GBD tissue (n = 5), GBD organoids at P5 (n = 7), PancD tissue (n = 3), and PancD organoids at P5 (n = 3); one‐way ANOVA. *P ≤ 0.05; **P ≤ 0.01; ***P ≤ 0.001; ****P ≤ 0.0001. If not otherwise indicated, comparisons between groups were not significantly different (*P* > 0.05). Abbreviations: APC, allophycocyanin; FITC, fluorescein isothiocyanate; UBC, ubiquitin C.

GBD samples were received as either whole GBDs or small 2‐cm^2^ tissue segments. Whole GBDs were drained of bile and the neck discarded. A longitudinal incision was used to expose the GBD mucosal surface. CBD and PancD samples were received intact and the wall of the ducts incised with a scalpel to expose the lumen. Tissues were washed three times with cold Hank’s balanced salt solution. Tissues were transferred to cold advanced (ADV)/Dulbecco’s modified Eagle’s medium (DMEM)‐F12 with 100 U/mL of penicillin and 100 μg/mL of streptomycin. The mucosal surface was then abraded with a scalpel to mechanically dissociate the epithelium (Fig. 1A). Dissociated epithelium was centrifuged at 300*g* for 5 minutes, washed twice and then either lysed for primary RNA or used for organoid derivation. For organoid derivation, epithelium was resuspended in growth factor reduced (GFR) Matrigel. Droplets (50 μL) were plated per well into a 24‐well plate. Matrigel was allowed to solidify for 5‐10 minutes at 37°C before adding 500 μL of ADV/DMEM‐F12 containing 1× N2 serum‐free supplement, 1× B27 serum‐free supplement, 2 mM of l‐glutamine, 100 U/mL of penicillin, and 100 μg/mL of streptomycin. The following growth factors were added: 20% R‐spondin conditioned media or 500 ng/mL of recombinant human R‐spondin 1 (RSPO‐1), 3 μM of CHIR 99021, 100 ng/mL of recombinant human Noggin, 2.5 μM of prostaglandin E2 (PGE_2_), 100 ng/mL of recombinant human epidermal growth factor (EGF), 5 μM of A 83‐01, and 10 μM of forskolin (FSK). For the first 48 hours, 10 μM of Y‐27632 was added to the media. Media was changed every 2‐3 days. Within 24‐48 hours, cystic organoid structures were observed. Organoids reached confluence after 5‐10 days and were split using the procedure below. Cells could be readily freeze/thawed using Cell Banker 2.

### Intrahepatic Bile Duct Isolation and Organoid Culture

Liver tissue (2 × 2 cm^2^) was frozen in Cell Banker 2 and stored at −80°C. Tissue was thawed at 37°C and then minced in a 10‐cm^2^ plate. Tissue fragments were placed in 20 mL of digestion media consisting of DMEM high glucose + GlutaMAX with 1% fetal calf serum (FCS), 100 U/mL of penicillin, 100 μg/mL of streptomycin, 128.4 μg/mL of dispase II, and 128.4 μg/mL of collagenase from *Clostridium histolyticum*. Tissue was digested for 4‐5 hours. Ducts were picked under a microscope, then placed in DMEM high glucose + GlutaMAX with 5% FCS. Ducts were centrifuged for 5 minutes at 200*g*. Ducts were resuspended in GFR Matrigel, and 50‐μL droplets were plated in a 24‐well plate, allowed to solidify at 37°C, and overlaid with 500 uL of one of two media: (1) extrahepatic media as described above with 10 μM of Y‐27632 or (2) intrahepatic isolation media for 3 days, after which the media was changed to intrahepatic expansion media as described.^(^ ^9^, ^14^ ^)^ See Supporting Table S2 for all reagents and manufacturers used in cell culture.

### Organoid Passaging

Extrahepatic organoids were split every 5‐10 days at a ratio of 1:2‐1:4. Organoids were incubated in cell recovery solution for 30 minutes at 4°C. Extrahepatic organoids were washed two times with ADV/DMEM‐F12 and pelleted at 300*g* for 4 minutes. Extrahepatic organoids were then dissociated with a p1000 pipette, resuspended in fresh Matrigel, and replated as above. The media was supplemented with 10 μM of Y‐27632 for 48 hours after splitting. Intrahepatic organoids were subjected to the same passaging procedure, except that cell recovery solution was not used.

### Immunocytochemistry, Flow Cytometry, and qPCR

See the Supporting Materials and Methods for details on immunocytochemistry, flow cytometry, qPCR, and RNA sequencing (RNA‐Seq). A list of antibodies and primers can be found in Supporting Tables S3 and S4.

### Statistical Analyses

Statistical analyses were carried out in Prism software (version 7; GraphPad Software Inc., La Jolla, CA). An unpaired *t* test or one‐way analyses of variance (ANOVAs) were performed followed by post‐hoc analyses, using either Dunnett’s, Tukey’s, or Sidak’s multiple comparisons tests.

## Results

### Derivation of Extrahepatic Bile Duct Organoids in Conditions Promoting WNT Signaling

**Fig. 2:**
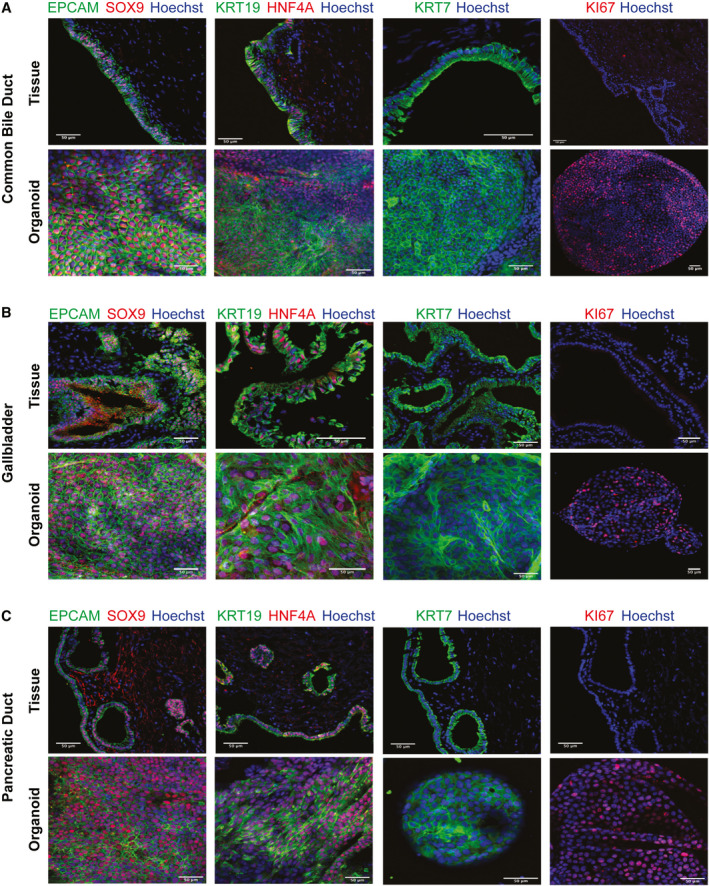
Extrahepatic tissues and organoids express biliary markers and show histological similarities. Representative IF images of EHBD organoids and tissues for selected markers. (A) CBD tissue and organoids. (B) GBD tissue and organoids (C) PancD tissue and organoids. Scale bar, 50 μm.

In the course of our previous experiments, we noted that extrahepatic cholangiocytes cultured as organoids (extrahepatic cholangiocyte organoids; ECOs) in the presence of RSPO‐1, dickkopf WNT signaling pathway inhibitor 1 (DKK‐1; a canonical WNT inhibitor), and EGF were capable of long‐term expansion, but did not express characteristic stem/progenitor markers such as LGR5 and PROM1.^(^ ^12^ ^)^ Thus, ECOs seemed to fundamentally differ from organoids derived from IHBDs, which do express these markers.^(^ ^9^ ^)^ In parallel, we also observed that growing ECOs in the presence of CHIR 99021, a small‐molecule inhibitor of glycogen synthase kinase 3 beta (GSK3β), resulted in lower levels of phosphorylated ß‐catenin and thus a higher level of WNT activity.^(^ ^12^ ^)^ Given these observations, we decided to test a culture media that promotes canonical WNT signaling. We hypothesized that this media would promote a progenitor phenotype in extrahepatic biliary organoids more closely resembling that of intrahepatic organoids. The resulting cells were designated biliary organoids, instead of cholangiocyte organoids, to underline this difference. Mechanical dissociation was used to isolate biliary epithelium from human CBD, GBD, and PancD (Fig. 1A), which allows the recovery of a near homogenous population of cells coexpressing the cholangiocyte markers, keratin (KRT) 7/KRT19 (94.7% ± 4.31%; mean ± SD; n = 2; Fig. 1B). The resulting cells were then grown in 3D Matrigel supplemented with RSPO‐1, CHIR 99021, Noggin, PGE_2_, EGF, A 83‐01, and FSK. Importantly, addition of both A 83‐01 (transforming growth factor [TGF‐β] inhibitor) and FSK (cAMP activator) were important for maintaining organoid cultures (Fig. 1C).^(^ ^9^ ^)^ Flow cytometry analyses on GBD and CBD, organoids demonstrated coexpression of KRT19 and KRT7 (95.04 ± 2.09% of cells; n = 5; Fig. 1D and Supporting Fig. S1). In total, seven CBD lines, nine GBD lines, and three PancD lines were derived in these conditions from donors ranging in age from 20 to 68 years (Supporting Table S1), with 100% of the tissue samples yielding organoids. Organoids displayed similar morphology and growth dynamics regardless of their tissue of origin and could not be readily distinguished from one another (Fig. 1A). Organoids from all three regions demonstrated a similar capacity for single‐cell clonality, with an average of 1.4% of single cells reforming organoids (Fig. 1E). In the absence of A 83‐01 and FSK, this was significantly reduced to 0.5% (Fig. 1C). qPCR demonstrated that EHBD organoids expressed adult stem/progenitor markers (LGR5 and PROM1), ductal markers (SRY‐box transcription factor 9 [SOX9], KRT7, and hepatocyte nuclear factor 1 beta [HNF1B]), and early hepatocyte markers (T‐box 3 [TBX3], hepatocyte nuclear factor 4 alpha [HNF4A]), but not the hepatocyte marker, albumin (ALB; Fig. 1F), and that the majority of these markers remained consistently expressed over time in culture (Supporting Fig. S2). Levels of ductal markers in EHBD organoids were comparable to or greater than tissue, with the exception of the mature biliary marker, HNF1B, which was reduced in EHBD organoids. The stem/progenitor markers, LGR5/PROM1 and TBX3, were significantly enriched in EHBD organoids compared to tissue. Immunofluorescence (IF) of Ki67 demonstrated that a substantial fraction of cells were actively proliferating (Fig. 2A‐C and Supporting Fig. S3). Interestingly, primary tissues had no detectable levels of Ki67 (Fig. 2A‐C and Supporting Fig. S4), suggesting that proliferation is extremely rare in the biliary epithelium. Finally, both organoids and tissues uniformly expressed epithelial cell adhesion molecule (EPCAM), SOX9, KRT19, HNF4A, and KRT7 (Fig. 2A‐C and Supporting Figs. S3 and S4). Taken together, these results demonstrate that biliary organoids can be derived from multiple, distinct regions of the extrahepatic biliary tree and that the promotion of canonical WNT signaling is associated with expression of stem/progenitor markers in these organoids.

### Transcriptomic Profiling of Primary Extrahepatic Biliary Epithelium Reveals Distinct Signatures Between Anatomical Regions

**Fig. 3:**
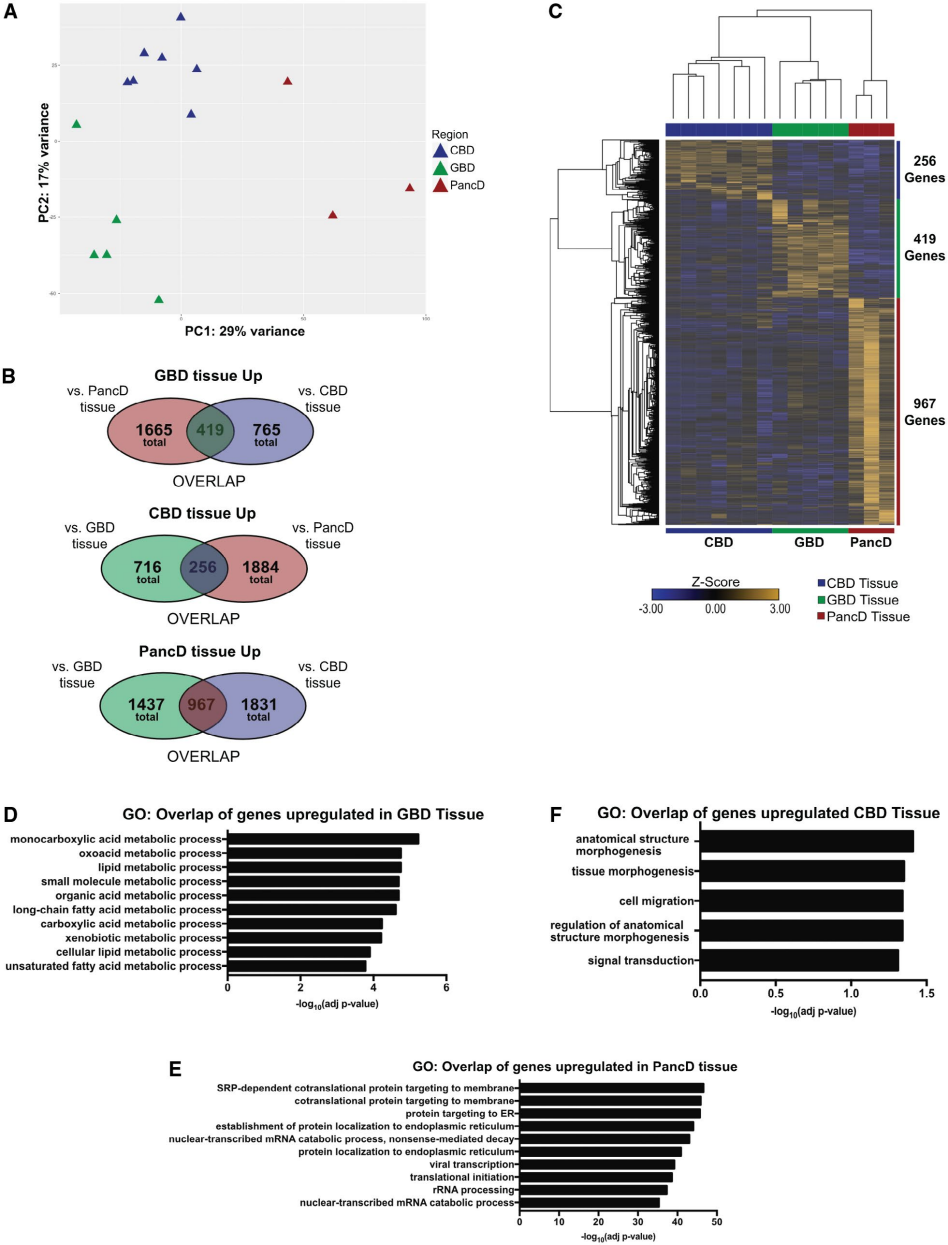
Gene expression profile of extrahepatic tissues uncovered regional‐specific markers and functions. (A) PCA of variance stabilized counts for the top 5,000 most variable genes between CBD (n = 7), GBD (n = 5), and PancD (n = 3) tissues. (B) Venn diagrams showing the number, as well as overlap, of genes up‐regulated in either GBD, CBD, or PancD tissues in comparison to each of the other two. (C) Heatmap of the tissue‐specific genes up‐regulated in CBD compared to both GBD and PancD tissues (n = 256 genes), GBD compared to both PancD and CBD tissues (n = 419 genes), and PancD compared to both CBD and GBD tissues (n = 967 genes). (D‐F) Top 10 most significant gene ontologies (biological process) for genes up‐regulated in GBD tissue compared to both CBD and PancD (D); PancD tissue compared to both CBD and GBD (E); only five terms were significantly enriched in CBD tissue compared to both GBD and PancD (F). Abbreviations: ER, endoplasmic reticulum; rRNA, ribosomal RNA.

To assess what differences may exist between organoids and their region of origin, we first decided to establish a transcriptional profile of primary CBD, GBD, and PancD epithelium. RNA‐Seq was performed on epithelial‐enriched samples from each tissue region. Of note, these epithelial cells were collected as described for organoid derivation without any purification step to avoid cellular stress associated with cell sorting. Thus, we cannot exclude limited contamination by nonbiliary cell types. Accordingly, hormonal markers could be detected in PancD samples (i.e., pancreatic polypeptide, somatostatin; Supporting File S1). Nonetheless, expression of biliary markers, such as KRT7, KRT19, and EPCAM, could be detected at a similar level across the three tissues, confirming enrichment of ductal epithelium. Based on this observation, we performed principal component analysis (PCA), which revealed clustering of samples by tissue type (Fig. 3A). Further, differential gene expression (DGE) analyses demonstrated that a number of genes were differentially expressed between tissues (Fig. 3B; Supporting File S1), thus uncovering regional‐specific markers. Overall, 419 genes were determined to be GBD specific, 967 PancD specific, and 256 CBD specific (Fig. 3B; Supporting File S2). In addition, we performed hierarchical clustering using these markers and show that both the samples and genes cluster by tissue, further validating their regional specificity (Fig. 3C). Gene Ontology (GO) analyses on tissue‐specific genes and also genes differentially expressed between pairs of tissues highlighted potential functional differences between the regions (a full list of GO terms for all comparisons below can be found in Supporting File S3). Of particular interest, GBD tissue was enriched in genes (i.e., UDP glucuronosyltransferase family 1 member A6 [UGT1A6], monoacylglycerol O‐acyltransferase 1 [MOGAT1], carbonic anhydrase 4, SRY‐box transcription factor 17 [SOX17], and vitamin D receptor [VDR]) involved in xenobiotic, lipid, carbohydrate, and steroid metabolism (Fig. 3D and Supporting Fig. S5A,B). Genes up‐regulated in PancD tissue (i.e., secretin receptor and caudal‐related homeobox 2 [CDX2]) were involved in protein targeting and localization to the membrane or endoplasmic reticulum (Fig. 3E and Supporting Fig. S5C,D). CBD‐specific genes (i.e., homeobox B2 [HOXB2], homeobox B3 [HOXB3], and ATP‐binding cassette subfamily A member 1 [ABCA1]) were involved in tissue morphogenesis and signal transduction (Fig. 3F and Supporting Fig. S5E,F). Finally, we looked at genes up‐regulated in both GBD and CBD, compared to PancD, tissue and found 856 overlapping genes (Supporting Fig. S5G,H; Supporting File S2). This list included genes involved in lipid, cholesterol, bile acid, and bilirubin metabolism (i.e., [FGF19], [NR1I2], VDR, UDP glucuronosyltransferase family 1 member A4, UGT1A6, UDP glucuronosyltransferase family 1 member A10, ABCA1, apolipoprotein A1, and peroxisome proliferator‐activated receptor gamma), all components of bile, which is only in contact with GBD/CBD epithelium and not the PancD. Taken together, these data suggest that the anatomical region could shape the expression profile of cholangiocytes within the biliary tree.

### Transcriptomic Profiling of Extrahepatic Biliary Organoids Reveals Down‐regulation of Cholangiocyte Markers and Up‐regulation of Cell‐Cycle and WNT Target Genes

**Fig. 4:**
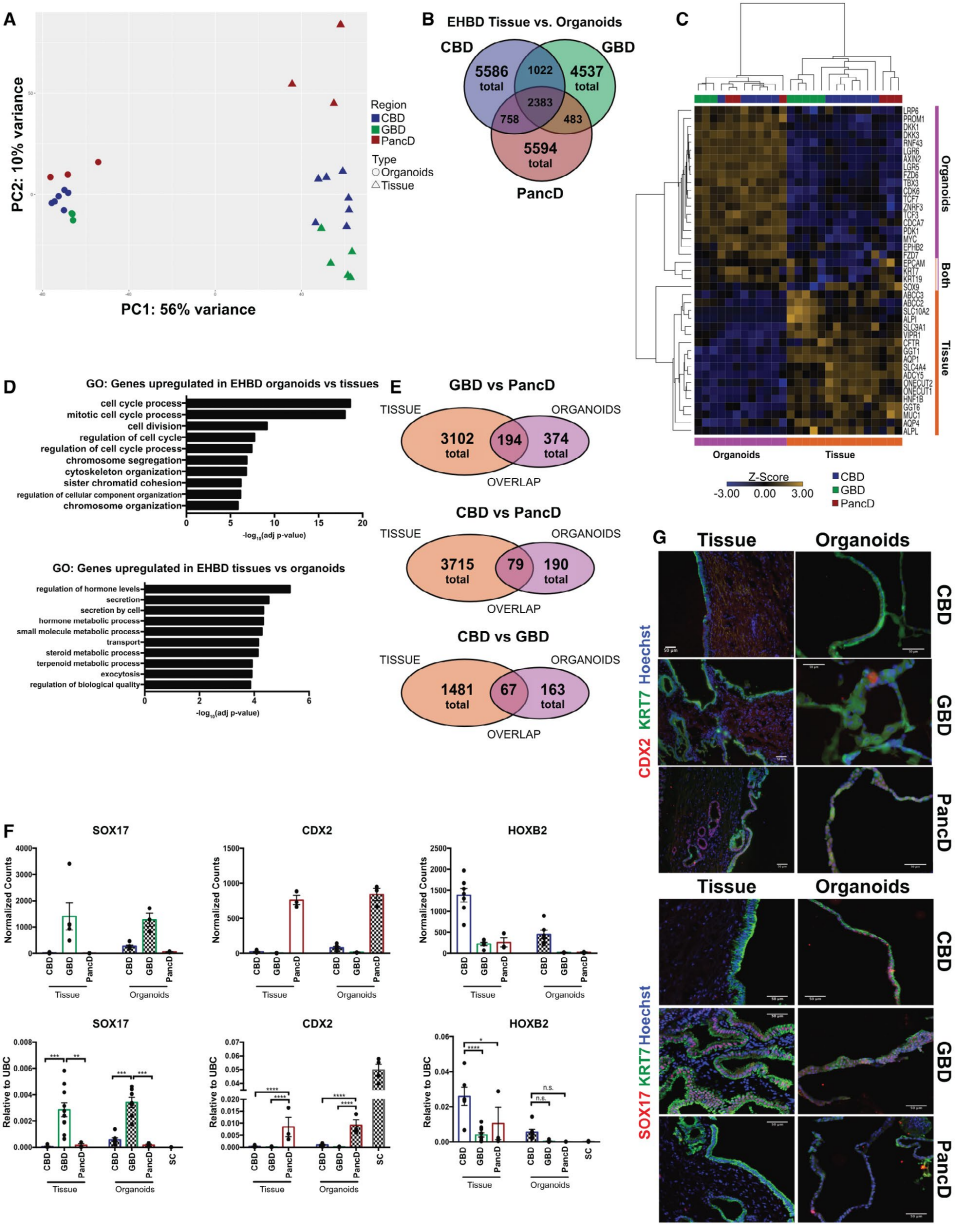
Comparison of the transcriptional profile of EHBD organoids and tissues suggest down‐regulation of functional biliary markers, up‐regulation of WNT and cell‐cycle pathways, and minimal maintenance of regional‐specific markers after *in vitro* culture. (A) PCA of variance stabilized counts for the top 5,000 most variable genes between CBD tissue (n = 7), CBD organoids (n = 6), GBD tissue (n = 5), GBD organoids (n = 3), PancD tissue (n = 3), and PancD organoids (n = 3). (B) Venn diagram showing the number, and overlap, of genes differentially expressed between CBD tissue and organoids, PancD tissue and organoids, or GBD tissue and organoids. (C) Heatmap of selected biliary and adult stem cell/WNT pathway genes. (D) Top 10 most significant gene ontologies (biological process) for the top 200 differentially expressed genes up‐regulated in extrahepatic tissues compared to organoids or up‐regulated in extrahepatic organoids compared to tissues. (E) Venn diagrams displaying number, as well as overlap, of genes differentially expressed comparing: GBD versus PancD tissues and organoids; CBD versus PancD tissues and organoids; and CBD versus GBD tissues and organoids. (F) RNA‐Seq normalized counts and qPCR validation of three genes found to retain tissue‐specific expression patterns in organoids. For qPCR validation: CBD tissue (n = 6), CBD organoids (n = 7), GBD tissue (n = 10), GBD organoids (n = 7), PancD tissue (n = 3), and PancD organoids (n = 3) and gene expression were normalized to UBC, and data were plotted as mean and SEM; one‐way ANOVA. Sigmoid colon (SC) organoids (n = 4) were used as controls. *P ≤ 0.05; **P ≤ 0.01; ***P ≤ 0.001; ****P ≤  0.0001; n.s. = not significant. (G) IF of EHBD organoids and tissues for two genes found to be tissue specific. Abbreviations: ABCC3, ATP‐binding cassette subfamily C member 3; ADCY5, adenylate cyclase 5; ALP1, alkaline phosphatase 1, isoform A; ALPL, alkaline phosphatase, biomineralization associated; AQP1, aquaporin 1; AQP4, aquaporin 4; CDCA7, cell division cycle‐associated 7; CDK6, cyclin‐dependent kinase 6; DKK3, dickkopf WNT signaling pathway inhibitor 3; EPHB2, ephrin type‐B receptor 2; FZD6, frizzled class receptor 6; FZD7, frizzled class receptor 7; LAP6, less adhesive pollen 6; ONECUT1, one cut homeobox 1; ONECUT2, one cut homeobox 2; PDK1, pyruvate dehydrogenase kinase 1; RNF43, ring finger protein 43; SLC4A4, solute carrier family 4 member 4; SLC9A1, solute carrier family 9 member A1; SLC10A2, solute carrier family 10 member 2; TCF3, transcription factor 3; UBC, ubiquitin C; ZNRF3, zinc and ring finger 3.

Having generated the transcriptional profile of the three regions of the extrahepatic biliary tree, we next investigated how EHBD organoids compare to their tissue of origin. To do so, we performed RNA‐Seq on passage 5 organoids, derived from CBD, GBD, and PancD, and compared their transcriptional profile to those of primary tissues. PCA revealed that the largest source of variation in the samples (56%) was between tissues and organoids, whereas tissue‐specific differences only accounted for 10% of variation between samples (Fig. 4A). Furthermore, DGE analyses showed that a large number of genes were differentially expressed between each organoid type and its corresponding tissue of origin (Fig. 4B; Supporting File S4). To further understand these differences, we examined the expression levels of selected genes involved in mature biliary function, adult stem/progenitors, WNT signaling, and cell cycle, which were differentially expressed between organoids and tissues (Fig. 4C). Hierarchical clustering of these genes highlighted the differences between organoids and tissues. We observed the induction of known WNT target genes (i.e., LGR5/6, AXIN2, minichromosome maintenance complex component 2, SRY‐box transcription factor 4, MYC proto‐oncogene [MYC], and transcription factor 7 [TCF7]), but also an increase in stem/progenitor markers not regulated by WNT (i.e., ATP‐binding cassette subfamily C member 1, actin filament‐associated protein 1, neural cell adhesion molecule, PROM1, TUB‐like protein 3, helicase, lymphoid specific, and chloride intracellular channel 4) shown previously to be expressed in intestinal stem cells and liver progenitor cells (Supporting Fig. S6E; Supporting File S5).^(^ ^15^, ^16^ ^)^ No induction of pluripotency genes (i.e., NANOG, octamer 4, and SRY‐box transcription factor 2) was observed. On the other hand, organoids displayed significantly lower expression of functional biliary markers (i.e., gamma‐glutamyltransferase [GGT] 1/6, cystic fibrosis transmembrane conductance regulator [CFTR], mucin 1, cell‐surface associated [MUC1], and vasoactive intestinal polypeptide receptor 1 [VIPR1]; Fig. 4C). Thus, our culture conditions, and the activation of WNT signaling, seem to promote a progenitor profile. Of note, ductal markers, such as KRT7, KRT19, EPCAM, and SOX9, were not differentially expressed between tissues and organoids, suggesting that these genes are intrinsic biliary markers and not affected by WNT or *in vitro* conditions. Furthermore, GO analyses of the top 200 differentially expressed genes (genes with the smallest false discovery rate) between organoids and their corresponding tissues were enriched for terms involved in cell‐cycle process and cell division. Genes up‐regulated in tissues compared to organoids were enriched for GO terms involved in secretion, transport, and metabolic processes (Fig. 4D and Supporting Fig. S7; Supporting File S6). We also assessed whether any regional tissue‐specific signatures were maintained in EHBD organoids. It was found that only 45% of significantly differentially expressed genes between CBD‐, GBD‐, and PancD‐derived organoids overlapped with differentially expressed genes previously identified in their corresponding primary tissues (Fig. 4E; Supporting Files S7 and S8). However, 91.6% of these genes showed the same directionality of expression when comparing organoid and tissue expression patterns. This suggests that EHBD organoids, despite differing from their tissue of origin, do maintain some regional‐specific markers in culture. Of note, expression of three of these genes—SOX17, CDX2, and HOXB2 (Fig. 4F,G and Supporting Fig. S8)—were validated using qPCR and/or IF. SOX17 was only expressed in GBD tissue whereas CDX2 and HOXB2 were exclusively expressed in PancD and CBD tissues, respectively. These expression patterns did not change significantly over time in culture (Supporting Fig. S9), although CBD organoids did show expression of SOX17 at the protein level despite minimal levels by qPCR (Fig. 4G and Supporting Fig. S8). Taken together, these data show that biliary epithelial cells lose, in part, their regional identity when grown as organoids in the presence of WNT signaling. Nonetheless, this “dedifferentiation” seems to be only partial given that expression of a few, but very specific, regional markers are maintained in EHBD organoids.

### Intrahepatic Bile Duct Organoids Cannot be Maintained in Extrahepatic Culture Conditions Promoting Canonical WNT

**Fig. 5:**
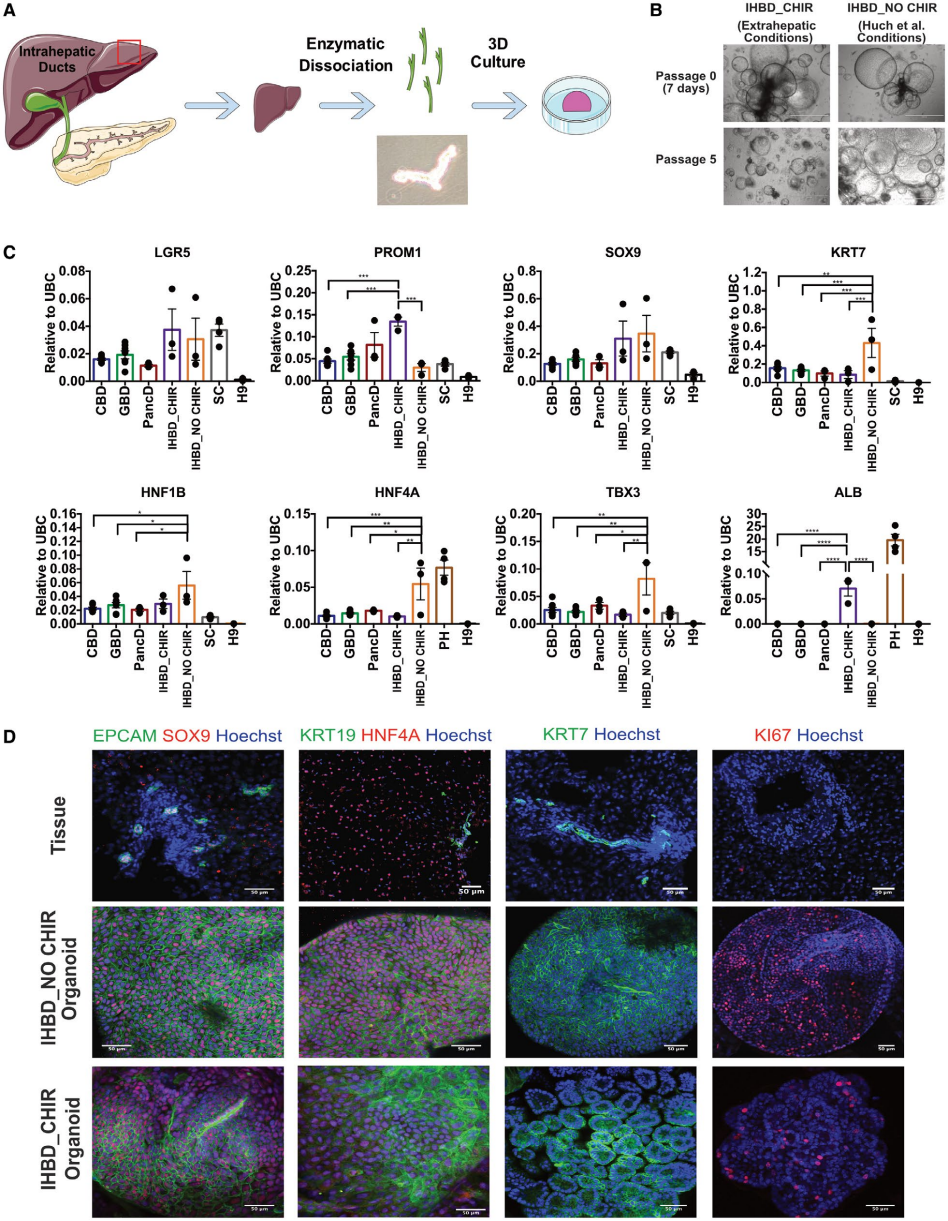
Intra‐ and extrahepatic organoids display divergent characteristics. (A) Diagram depicting the isolation procedure for IHBDs and image showing an isolated duct. (B) Images showing IHBD organoids grown in either Huch et al. conditions (IHBD_NO CHIR) or extrahepatic culture conditions (IHBD_CHIR) at passage 0 or at passage 5. Scale bars, 1,000 μm. (C) qPCR of IHBD_CHIR (n = 3) and IHBD_NO CHIR (n = 3) organoids in comparison to EHBD organoids from CBD (n = 7), GBD (n = 7), and PancD (n = 3) at passage 5. Primary hepatocytes (PH; n = 4), sigmoid colon organoids (SC; n = 4), and H9 embryonic stem cells (H9; n = 3) were used as controls. Gene expression was normalized to UBC, and data were plotted as mean and SEM. *P ≤ 0.05; **P ≤ 0.01; ***P ≤ 0.001; ****P ≤  0.0001. If not otherwise indicated, comparisons between groups were not significantly different (*P* > 0.05); one‐way ANOVA. (D) IF of IHBD_CHIR, IHBD_NO CHIR, and primary liver tissue for selected markers. Scale bars, 50 μm. Abbreviation: UBC, ubiquitin C.

**Fig. 6:**
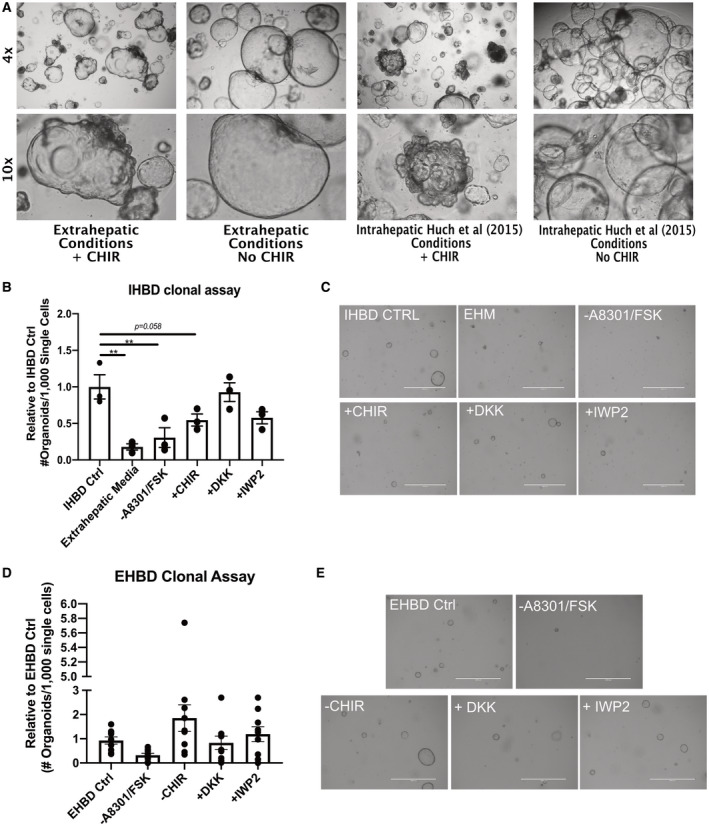
Intra‐ and extrahepatic organoids display differences in morphology, proliferation, and self‐renewal in response to GSK3β inhibition. (A) Images of IHBD_NO CHIR organoids transferred into either extra‐ or intrahepatic media (as described by Huch et al., 2015), with or without the addition of 3 μM of CHIR 99021. (B) Number of organoids formed per 1,000 viable single cells plated from IHBD_NO CHIR organoids that were cultured for 7 days in either intrahepatic media (IHBD Ctrl), extrahepatic media containing CHIR 99201 (EHM), intrahepatic media without A 8301 or FSK (‐A8301/FSK), intrahepatic media with 3 μM of CHIR 99201 (+CHIR), intrahepatic media with 100 ng/mL of DKK1 (+DKK1), or intrahepatic media with 2.5 μM of IWP2 (+IWP2). Data were plotted as mean and SEM and normalized relative to IHBD ctrl conditions (n = 3 independent experiments, n = 1 IHBD donor cell line; one‐way ANOVA). (C) Images of IHBD_NO CHIR organoids following 7 days of culture for the clonality assay. (D) Number of organoids formed per 1,000 viable single cells plated from EHBD organoids that were cultured for 7 days in either extrahepatic media containing CHIR 99021 (EHBD ctrl), extrahepatic media without A 8301 or FSK (‐A8301/FSK), extrahepatic media without CHIR 99201 (‐CHIR), extrahepatic media with 100 ng/mL of DKK1 (+DKK1), or extrahepatic media with 2.5 μM of IWP2 added (+IWP2). Data were plotted as mean and SEM and normalized relative to EHBD ctrl (n = 9 independent experiments, n = 3 EHBD donor cell lines, one per region; one‐way ANOVA). (E) Images of EHBD organoids following 7 days of culture for the clonality assay. **P ≤ 0.01, if not otherwise indicated, comparisons between groups were not significantly different (*P *> 0.05). Abbreviation: Ctrl, control.

We next sought to establish organoids from IHBDs using the same culture conditions used to derive EHBD organoids. For that, liver tissue obtained from deceased transplant organ donors was subjected to enzymatic dissociation, and the resulting cells were grown in the conditions described above (Fig. 5A). The resulting organoids (IHBD_CHIR) grew well for the first 2‐3 weeks. Interestingly, IHBD_CHIR organoids were morphologically distinct from EHBD organoids (Supporting Fig. S10A), displaying a heterogeneous appearance, with some organoids having cryptic/budding structures, whereas others maintained a cystic structure similar to EHBD organoids. After five to seven passages, these cryptic structures began to predominate, proliferation ceased, and cultures could not be maintained. Given this result, we also isolated IHBD organoids in conditions published by Huch et al.^(^ ^9^ ^)^ The resulting organoids (IHBD_NO CHIR) maintained their proliferative ability for at least 10 passages without evidence of deterioration (Fig. 5B) while displaying a homogenous cystic morphology. qPCR analyses of EHBD, IHBD_CHIR, and IHBD_NO CHIR organoids revealed only minor differences in expression of biliary and stem/progenitor markers (Fig. 5C). IF confirmed that, similarly to EHBD organoids, IHBD organoids expressed EPCAM, SOX9, KRT19, and KRT7 (Fig. 5D and Supporting Fig. S11). Of note, IHBD organoids did express HNF4A whereas this transcription factor was absent from primary IHBD tissue. Ki67 expression was present in the majority of cells in IHBD_NO CHIR organoids, but was variable in IHBD_CHIR organoids, and absent in primary tissue (Fig. 5D and Supporting Fig. S10B). Additional investigations revealed that the presence of CHIR 99021 in media was likely responsible for the divergence between IHBD_CHIR and IHBD_NO CHIR organoids (Fig. 6A and Supporting Fig. S12).

Established IHBD_NO CHIR organoids were dissociated into single cells and transferred into EHBD media containing CHIR 99021 or into IHBD media containing CHIR 99201, IWP2, DKK, or IHBD media without A 83‐01/FSK (Fig. 6B,C). Promoting canonical WNT or TGFβ pathways significantly reduced the clonality of IHBD_NO CHIR organoids, suggesting that these factors are detrimental to the self‐renewal of intrahepatic biliary cells. Similar experiments were performed with EHBD organoids, and the presence or absence of CHIR 99021 had little effect on their clonality. Interestingly, inhibition of WNT signaling with IWP2 or DKK had little impact on either IHBD_NO CHIR or EHBD organoids (Fig. 6D,E and Supporting Fig. S10C), suggesting that the canonical WNT pathway is not necessary for cholangiocytes to proliferate *in vitro*. Taken together, these data demonstrate that cells from the extra‐ and intrahepatic biliary tree could diverge in their response to canonical WNT. Indeed, continuous activation of canonical WNT signaling appears detrimental to the proliferation and self‐renewal of IHBD, but not EHBD, organoids. However, this difference seems to have little consequence on the expression of biliary markers.

### Intra‐ and Extrahepatic Organoids Display Different Transcriptional Profiles

**Fig. 7:**
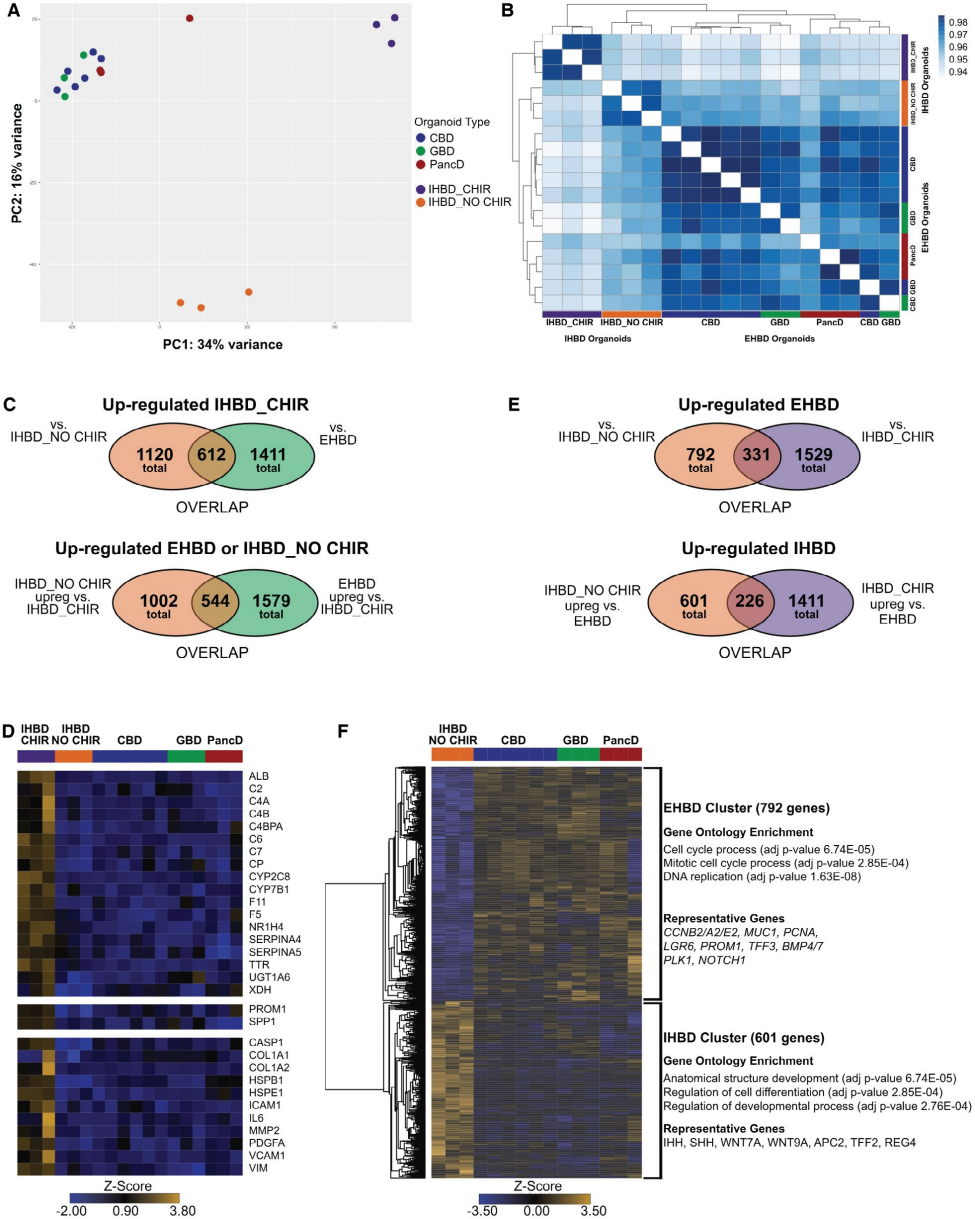
Extra‐ and intrahepatic organoids are transcriptionally unique. (A) PCA of variance stabilized counts for the top 5,000 most variable genes between CBD (n = 6), GBD (n = 3), and PancD (n = 3) organoids as well as IHBD organoids cultured in extrahepatic conditions (IHBD_CHIR, n = 3) or in conditions described by Huch et al. (IHBD_NO CHIR, n = 3). (B) Sample distance matrix of the Pearson correlation between EHBD and IHBD organoids. (C) Venn diagram showing the number, and overlap, of genes differentially expressed in IHBD_CHIR organoids compared to IHBD_NO CHIR and EHBD organoids. (D) Heatmap of selected genes up‐regulated in IHBD_CHIR organoids. (E) Venn diagram showing the number, and overlap, of genes differentially expressed between EHBD organoids compared to the two IHBD organoid types. (F) Heatmap of the 1,393 genes differentially expressed between EHBD and IHBD_NO CHIR organoids as well as representative GO terms (biological process) and examples of genes up‐regulated in each organoid type. Abbreviations: APC2, APC regulator of WNT signaling pathway 2; BMP4/7, bone morphogenetic proteins 4 and 7; C2, complement C2; C4A, complement C4A; C4B, complement C4B (Chido blood group); C4BPA, complement component 4 binding protein alpha; C6, complement C6; C7 complement C7; CCNB2/A2/E2, cyclin B2/A2/E2; COL1A1, collagen type I alpha 1 chain; COL1A2, collagen type I alpha 2 chain; CP, ceruloplasmin; CYP2C8, cytochrome P450 family 2 subfamily C member 8; F5, coagulation factor V; F11, coagulation factor XI; HSPB1, heat shock protein beta 1; HSPE1, heat shock protein family E (Hsp10) member 1; ICAM1, intercellular adhesion molecule 1; IL6, interleukin 6; LGR6, leucine‐rich repeat–containing G‐protein‐coupled receptor 6; MMP2, matrix metalloproteinase 2; PDGFA, platelet‐derived growth factor subunit A; PLK1, polo‐like kinase 1; REG4, regenerating family member 4; SERPINA4, serpin family A member 4; SERPINA5, serpin family A member 5; TFF2, trefoil factor 2; TFF3, trefoil factor 3; WNT7A, Wnt family member 7A; WNT9A, Wnt family member 9A; XDH, xanthine dehydrogenase.

In addition to performing the basic characterizations of our EHBD and IHBD organoids, we also performed RNA‐Seq comparisons on CBD, GBD, PancD, IHBD_CHIR, and IHBD_NO CHIR organoids. PCA demonstrated that the largest source of variation was between EHBD and IHBD organoids. EHBD organoids clustered closely together regardless of their tissue of origin, whereas both IHBD_CHIR and IHBD_NO CHIR clustered separately from EHBD organoids and each other (Fig. 7A). Correlation analysis further confirmed this (Fig. 7B). Given that all of the EHBD organoid types clustered closely and the number of genes differentially expressed between the three organoid types was minimal in our earlier analyses, we combined them into a single “EHBD” group for further analyses. We then sought to understand the transcriptional divergence between IHBD_CHIR, IHBD_NO CHIR, and EHBD organoids. A total of 2,122 genes were differentially expressed between IHBD_CHIR and IHBD_NO CHIR organoids, whereas 2,990 genes were differentially expressed between IHBD_CHIR and EHBD organoids. Of note, 544 genes were up‐regulated in both IHBD_NO CHIR and EHBD organoids compared to IHBD_CHIR organoids (Fig. 7C; Supporting File S9). As expected, given the proliferation differences observed between the organoids, these genes were enriched significantly for two GO terms, including mitotic cell‐cycle process (adjusted *P* value = 4.01E10^−2^) and cell‐cycle process (adjusted *P* value = 4.06E10^−2^). Interestingly, 612 genes were up‐regulated in IHBD_CHIR organoids compared to both IHBD_NO CHIR and EHBD organoids. These genes included hepatocyte (i.e., ALB, transthyretin [TTR], nuclear receptor subfamily 1 group H member 4 [NR1H4], cytochrome P450 family 7 subfamily B member 1 [CYP7B1], and complement/clotting factors), stem/progenitor (i.e., PROM1, secreted phosphoprotein 1 [SPP1]), and cell death/senescence/epithelial mesenchymal transition (i.e., caspase‐1 [CASP1], vimentin [VIM]) markers (Fig. 7D). Thus, this analysis suggests complex differences between these organoids without uncovering a major source of divergence.

We next sought to more broadly understand the differences between IHBD and EHBD organoids. Given the transcriptional signature of the IHBD_CHIR organoids and their known proliferative difference, we focused these comparisons on EHBD and IHBD_NO CHIR organoids. A total of 1,393 genes were differentially expressed between EHBD and IHBD_NO CHIR organoids (Fig. 7E). The 601 genes up‐regulated in IHBD_NO CHIR organoids were enriched for GO terms involved with developmental processes and cell differentiation (Fig. 7F; Supporting File S10) Interestingly, these genes included the hedgehog pathway ligands, Indian hedgehog (IHH) and Sonic hedgehog (SHH), which have been shown to promote the maintenance of intrahepatic progenitor cells.^(^ ^17^ ^)^ The 792 genes up‐regulated in EHBD organoids were enriched for GO terms involved in cell‐cycle process and DNA replication, including genes such as cyclin B2/A2/E2 and proliferating cell nuclear antigen (PCNA). This finding could suggest that proliferation could be controlled by different mechanisms between intra‐ and extrahepatic cholangiocytes. Such differences could have broad implications, given that cholangiopathies are associated with cholangiocyte senescence, and thus proliferative capacity could play an essential role in disease development.^(^ ^18^ ^)^ In sum, these results demonstrate that EHBD and IHBD organoids could be transcriptionally distinct. However, this divergence may not necessarily be associated with their region of origin and could be induced by different response to WNT and/or different culture conditions.

### Extrahepatic Organoids do not Demonstrate Differentiation Capacity Toward a Hepatocyte Fate

**Fig. 8:**
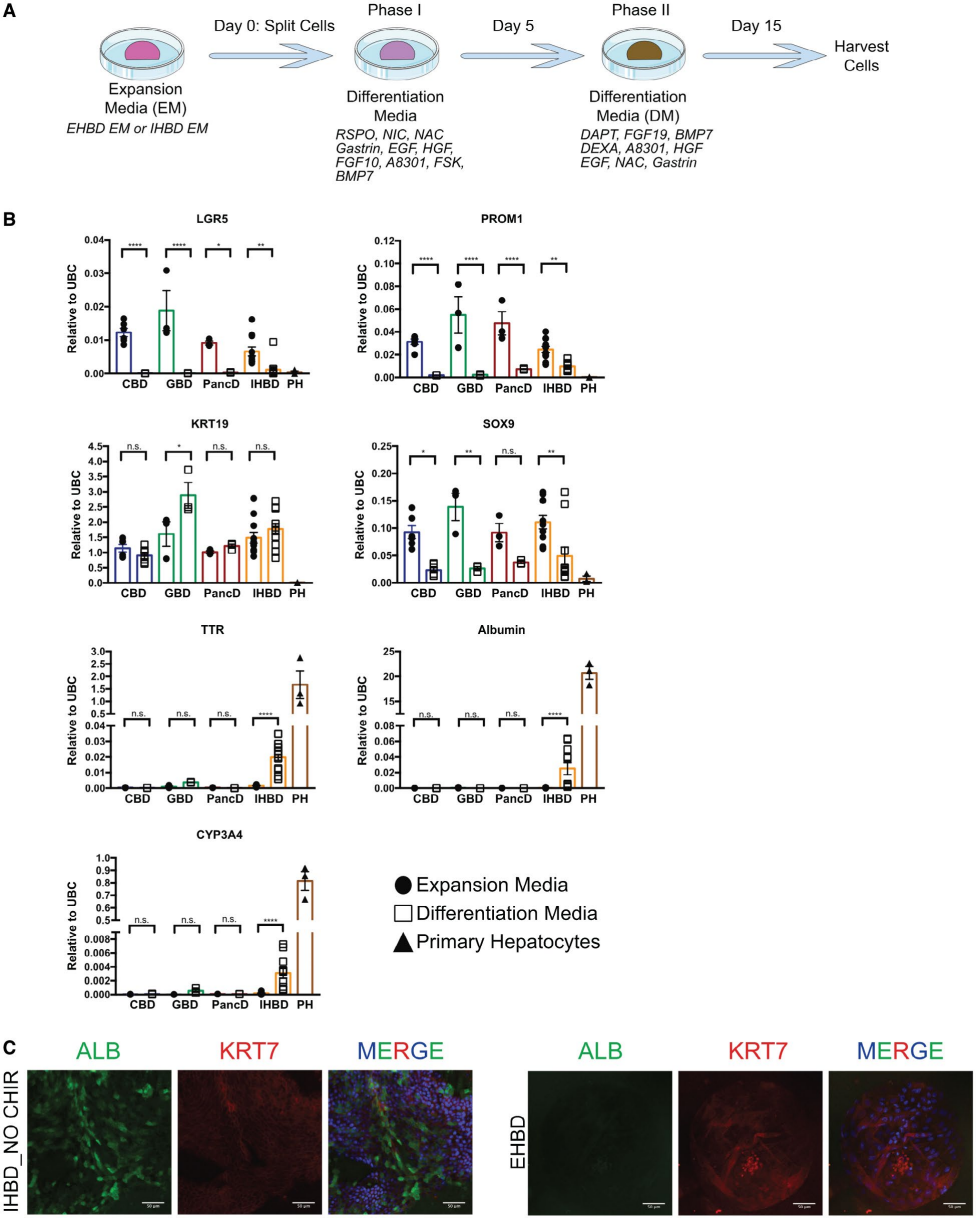
Extrahepatic organoids do not display differentiation capacity toward a hepatocyte fate when compared to intrahepatic organoids. (A) Diagram depicting the differentiation protocol. (B) qPCR showing expression of biliary and hepatocyte markers in IHBD_NO CHIR, CBD, GBD, or PancD organoids grown in expansion media (EM) or differentiation media (DM; n = 3‐11 independent experiments, n = 1‐3 donors per tissue region). Primary hepatocytes (PH; n = 3) were used as controls. Gene expression was normalized to UBC, and data were plotted as mean and SEM. *P ≤ 0.05; **P ≤ 0.01; ***P ≤ 0.001; ****P ≤ 0.0001. (C) IF of IHBD_NO CHIR and EHBD organoids in DM conditions for ALB and KRT7. Abbreviations: BMP7, bone morphogenetic protein 7; DAPT, dual antiplatelet therapy; DEXA, dual‐energy x‐ray absorptiometry; FGF19, fibroblast growth factor 19; HGF, hepatocyte growth factor; NAC, N‐acetylcysteine; NIC, nicotinamide; n.s., not significant; RSPO, R‐spondin; UBC, ubiquitin C.

Our gene expression analyses revealed that IHBD organoids might differ from EHBD organoids in their capacity to display a background expression of hepatocyte markers. Thus, we decided to validate this observation by assessing the differentiation capacity of EHBD versus IHBD organoids. For that, we used the differentiation protocol published by Huch et al. (Fig. 8A). As reported, under differentiation conditions, IHBD_NO CHIR organoids showed significantly reduced expression in stem/progenitor markers, suggesting differentiation (Fig. 8B). However, KRT19 remained stably expressed, suggesting that biliary identity was not completely lost. qPCR induction of a low‐level expression of hepatocyte‐specific markers, such as TTR, cytochrome P450 family 3 subfamily A member 4 (CYP3A4), and ALB, was also observed in IHBD_NO CHIR organoids. However, this was significantly lower than levels expressed in primary human hepatocytes. IHBD_CHIR organoids demonstrated identical trends (Supporting Fig. S13A), indicating that the conditions in which IHBD organoids were isolated and maintained did not impact their ability to express hepatocyte‐specific markers.

We next assessed the effect of this differentiation protocol on EHBD organoids from all three tissue regions. When subjected to the differentiation protocol, EHBD organoids, similarly to IHBD organoids, had significantly decreased expression of LGR5, PROM1, and SOX9, with stable expression of KRT19 (Fig. 8B). However, EHBD organoids showed no induction of the hepatocyte‐specific markers, TTR, ALB, or CYP3A4. These results were confirmed at the protein level, showing ALB expression exclusively in IHBD organoids (Fig. 8C and Supporting Fig. S13B). Given the negative results for EHBD organoids, we decided to screen different culture conditions capable of inducing expression of hepatocyte‐specific markers. We tested a wide variety of growth factors and small‐molecule inhibitors aimed at perturbing signaling pathways important in liver development and regeneration (Supporting Table S5, Supporting Fig. S14A‐C, and data not shown). None of these compounds significantly increased the expression of hepatocyte‐specific markers in EHBD organoids. These results suggest that only biliary cells located within the liver have the capacity to express hepatocyte markers and reveal a fundamental divergence in terms of the capacity of differentiation between different regions of the biliary tree.

## Discussion

It has long been known that human cholangiopathies show regionalization, with many of these diseases targeting large EHBDs early in disease pathogenesis. Here, we established a transcriptional profile of epithelial enriched samples from CBD, GBD, and PancD tissues. Of note, the RNA‐Seq data for these tissues were limited by our isolation technique, and the presence of nonbiliary epithelial cells in the samples cannot be ruled out. Despite this limitation, we were able to assess expression differences between the three tissues, gain insight into functional differences between the tissues, and identify regional tissue‐specific markers such as HOXB2, CDX2, and SOX17. Most notably, GBD tissue displayed higher expression of genes associated with active metabolic processes involved in bile modification, including xenobiotic, lipid, and cholesterol metabolism. Our data suggest that each of these three tissue regions display a transcriptional profile in line with their function in bile transport, while maintaining a key underlying expression of essential cholangiocyte‐specific genes (i.e., CFTR, HNF1B, KRT7, and KRT19). Of these three tissues, only GBD tissue has ever been previously sequenced.^(^ ^19^ ^)^ Kampf et al. performed RNA‐Seq on GBD tissue as part of the Human Protein Atlas. In this work, the transcriptome of GBD tissue was compared to 27 other human tissues, but did not include CBD or PancD. Our own results appear to be in line with their findings, given that many genes identified in our own data set as GBD tissue specific (i.e., ATP‐binding cassette subfamily C member 2 [ABCC2], SPP1, forkhead box L1, and MOGAT1) were also identified by Kampf et al. This lends further support to our conclusions that tissue‐specific differences exist between these three extrahepatic regions. Nonetheless, future research is needed to understand whether these differences originate from the existence of different cholangiocytes and whether cellular diversity could play a role in the development of cholangiopathies.

In addition to profiling extrahepatic biliary tissues, we established and characterized organoids from different regions of the biliary tree. EHBD organoids were found to be transcriptionally distinct from their tissue of origin, increasing expression of proliferative and adult stem/progenitor‐specific markers *in vitro* while down‐regulating some functional biliary markers. This finding is contrary to other epithelial organoid systems, such as intestinal organoids, which have been shown to maintain a high degree of similarity and function to their tissue of origin *in vitro*.^(^ ^20^ ^)^ This finding is also contrary to our previously described ECOs, which were cultured in the presence of DKK‐1, a canonical WNT pathway inhibitor. ECOs, unlike the currently described EHBD organoids, maintained an expression profile similar to their tissue of origin and did not express adult stem/progenitor markers such as LGR5 or PROM1.^(^ ^12^ ^)^ On the other hand, adding GSK3β inhibitor augments the expression of stem/progenitor markers in extra‐ and intrahepatic organoids, suggesting that an increase in the WNT signaling pathway could promote a progenitor‐like identity. However, a number of these markers are also downstream of the WNT pathway, and thus their expression might not be a functional demonstration of a stem/progenitor cell state. Accordingly, EHBD organoids do not acquire the competence to differentiate into hepatocytes even after prolonged culture in the presence of a GSK3β inhibitor. WNT signaling therefore might only control the expression of a specific set of markers, which by themselves are not sufficient to establish multipotency. This observation reinforces recent single‐cell transcriptomic studies, showing that intrahepatic cholangiocytes neither express canonical WNT signaling markers, including Lgr5, nor do they rely on canonical WNT signaling for ductular reaction.^(^ ^21^, ^22^, ^23^ ^)^ Thus, expression of Lgr5, which is commonly used to characterize adult stem cells *in vitro*, might not mark intrahepatic biliary progenitors *in vivo*. Similarly, WNT canonical signaling might not be necessary for cholangiocyte activation in chronic biliary injuries. Of note, we have previously shown that noncanonical WNT is active in ECOs grown in the presence of DKK1, likely through mechanisms involving R‐spondin, which is an essential additive of the culture system.^(^ ^12^, ^13^ ^)^ Similarly, a report has suggested a function for noncanonical WNT in ductular reactions in the mouse.^(^ ^21^ ^)^ However, these findings remain controversial given that inhibition of WNT/planar cell polarity signaling does not affect intrahepatic cholangiocyte proliferation upon injury in the mouse and the role of this pathway seems to be related more to profibrotic cytokine production.^(^ ^24^ ^)^ Even less is known about these processes in the extrahepatic biliary tree. Thus, future research should examine the mechanisms underpinning our observations *in vitro*, as well as what role canonical versus noncanonical WNT signaling may play in the extrahepatic biliary tree *in vivo*.

Within the intrahepatic biliary compartment, several reports have demonstrated that intrahepatic bile ducts likely contain a population of bipotent progenitor cells capable of differentiating into hepatocyte‐like cells after chronic injuries *in vivo*.^(^ ^9^, ^25^ ^)^ There have also been reports that, in the EHBDs and GBD, biliary cells may also be capable of differentiating to hepatocyte‐like cells.^(^ ^26^, ^27^ ^)^ However, organoids derived from these two compartments have never been directly compared before. Here, we show that IHBD‐ and EHBD‐derived organoids cannot be grown in the exact same culture condition in the presence of GSK3β inhibitor. Indeed, canonical WNT signaling caused IHBD organoids to cease proliferation whereas EHBD could be grown over a prolonged period of time in the presence of GSK3β inhibitor. This difference in culture requirement could explain, in part, the divergent transcriptional profiles uncovered by our analyses and also the different capacity for expressing hepatocytes markers. Indeed, it has been shown previously that WNT produced by macrophages could drive intrahepatic liver progenitor cells toward a hepatocyte fate during tissue injury.^(^ ^28^ ^)^ Thus, the effect of CHIR 99021 on IHBD organoids could be reminiscent of these mechanisms. This divergence may originate from the embryonic origin of each of these tissues. Indeed, the CBD, GBD, and PancD are derived from a common pancreatobiliary progenitor,^(^ ^3^ ^)^ whereas IHBDs are derived from bipotent hepatoblasts, which give rise to hepatocytes and intrahepatic cholangiocytes.^(^ ^3^ ^)^ This embryonic origin could facilitate transdifferentiation or dedifferentiation/differentiation of IHBD organoids between the two lineages and could also explain the expression of specific markers in IHBD organoids.

In conclusion, the results presented here have demonstrated that regional diversity exists in the human biliary tree and that this regionalization could be lost, in part, after *in vitro* culture. In addition, only IHBD organoids seem able to acquire, in specific conditions, the capacity to express hepatocyte markers. Thus, fundamental differences exist between intra‐ and extrahepatic biliary epithelia, which are not erased by *in vitro* culture and which are defined by their anatomical location and also their embryonic origin. These results may provide insight into understanding the regionalization of human cholangiopathies. Finally, it is important for future research to examine what role the anatomical niche occupied by these different biliary cell types may play in shaping their expression profile, given that it may control regenerative processes during liver and/or biliary disease.

## Author Contributions

C.A.R.: study design, execution of experiments and collection of data, interpretation of data, figure production, and manuscript writing. S.G.T. and C.M.M.: execution of experiments (maintenance of IHBD lines, IHBD differentiation experiments, and data collection and analysis). W.Y.L. and S.J.F.: derivation and expansion of IHBD lines in extrahepatic culture conditions, provision of human liver tissue samples. S.E.A.: execution of experiments (derivation and maintenance of EHBD cell lines, qPCR data collection, and analysis for EHBD differentiation screening experiments). N.G., F.S., B.W., and O.T.: collection and preparation of extrahepatic tissue samples. R.A.T., F.O.C., J.R.F., D.M., and T.G.M.: RNA‐Seq data collection, processing, and/or analysis. R.L.G.: bioinformatics support, manuscript editing for important intellectual contributions. A.R. J.M.K. and M.Z.: primary colon tissue and RNA sample provision, ethical approvals, editing and final approval of the manuscript. N.R.F.H.: early study supervision and manuscript editing for important intellectual content. G.C.O. and K.S.P.: primary tissue provision, ethical approvals, and editing and final approval of the manuscript. T.A.W.: study supervision, editing and final approval of the manuscript. L.V.: design and concept of study, study supervision, interpretation of data, and editing and final approval of manuscript.

## Supporting information

Supplementary Material

File S1

File S2

File S3

File S4

File S5

File S6

File S7

File S8

File S9

File S10
