# Supplementary material for: Regional Differences in Human Biliary Tissues and Corresponding In Vitro–Derived Organoids
Source: Hepatology. 2021 Feb 6;73(1):247–67. doi: 10.1002/hep.31252 (PMC8641381; doi:10.1002/hep.31252)
Supplement: Supplementary file 1 — Supplementary Material [file HEP-73-247-s006.pdf]

Supplementary Materials and Methods

Immunocytochemistry

Whole mount organoids

Organoids were grown on 9 mm round glass coverslips (VWR) and then washed for 5 min in PBS, fixed in 4% paraformaldehyde (PFA) for 20 min at room temperature (RT), and blocked/permeabilized for 3 h in 10% donkey serum and 0.1% Triton-X. Coverslips were stained as previously described (1), except secondary antibodies were applied overnight at 4°C. Coverslips were removed using forceps and mounted on glass slides using Fluoromount G (ebioscience). A list of antibodies used can be found in **Supplementary Table 3**.

Tissue and OCT-embedded organoids

Tissue samples were fixed on ice for 30-45 min in 4% PFA. Tissues were then cryoprotected in 30% sucrose overnight on ice. Organoids were removed from matrigel using cell recovery solution and fixed for 20 min in 4% PFA and washed twice with PBS. Tissue and organoids were then embedded in OCT, snap frozen, and sectioned at 8 µm. Slides were blocked with 10% donkey serum/0.1% Triton-X for 1 h. Primary antibodies, diluted in 1% donkey serum/0.1% Triton-X (antibody diluent), were applied overnight at 4°C. Slides were washed three times for 5 min with antibody diluent. Secondary antibodies in antibody diluent were applied for 1h at RT. Slides were washed three times with antibody diluent, nuclei counter-stained with Hoechst 33258, and mounted with Fluoromount G. All images were acquired using either a Zeiss LSM700 laser scanning confocal or a Leica DMLB fluorescent microscope and analyzed using ImageJ.

Flow cytometry

Primary epithelium and/or organoids were dissociated to single cells using TrypLE Express. Cell staining and flow cytometry were performed as previously described (1).

Quantitative RT-PCR (qPCR)

Total RNA was extracted using either the RNeasy Mini or Micro Kit (Qiagen) depending on the expected RNA yield. Total RNA was reverse transcribed using Superscript II Reverse Transcriptase (Invitrogen). 10µL reactions were prepared using the SensiMix SYBR Low-ROX Kit (Bioline) and run on either a Life Technologies QuantStudio 12K Flex or QuantStudio 6 machine in technical duplicate. All genes were normalized to the housekeeping gene Ubiquitin (UBC). Primer sequences are listed in **Supplementary Table 4**.

Primary human controls for qPCR

Human hepatocytes were purchased from Biopredic International, France. *Human sigmoid colon organoids* were derived from biopsy samples and cultured as described previously (2). *Human embryonic stem cells* were cultured as previously described and the line H9 used for all experiments (3).

Single Cell Clonality Assays

Extrahepatic and intrahepatic bile duct organoids (IHBD_NO CHIR) were removed from matrigel and dissociated to single cells. Organoids were incubated at 4°C in cell recovery solution for 30 minutes to remove cells from the matrigel. Organoids were then incubated for 5 minutes in accutase to dissociate the organoids to single cells. Cells were washed once and pelleted at 300 x g. Viable cells were counted with tryphan blue and 1,000 single cells were re-seeded per well in matrigel. Single cells were then cultured for 7 days. The number of organoids formed in each well were counted on day 7.

RNA-Sequencing

RNA was isolated from 3-7 biological replicates from organoids at Passage 5 or mechanically dissociated extrahepatic epithelial enriched tissues as described above. RNA samples were sequenced at the NIAID Genomics Technologies Section. Poly-A purified mRNA truSeq libraries were prepared using the Illumina NeoPrep system. Single-end 76bp reads were obtained using a NextSeq500. At least 20 million reads/sample evenly distributed across the lanes were obtained. If this number of reads was not reached, the samples were re-sequenced and sequencing replicates merged. Reads were trimmed to 70 bp and reads less than 40 bp were discarded. Transcript abundance was estimated using *Salmon* with default settings (4). *Tximport* was used to summarize transcript abundances to the gene level and *DeSeq2* was used for principal component and differential gene expression analyses in R (5,6). Only protein coding genes in the Ensembl BioMart GRCh38 database were included in analyses. A significance cut-off for differentially expressed genes was set as False Discovery Rate (FDR) less than 0.05 and a $\log_2$FoldChange with an absolute value greater than 1.

Heatmaps of the variance stabilized transformed counts were generated using Morpheus (https://software.broadinstitute.org/morpheus). Hierarchical clustering was performed using one minus Pearson correlation with average linkage. Gene ontology (GO) enrichment analyses were performed using GOrilla with a significance cut off for enriched GO terms set as a FDR less than 0.05 using the Benjamini and Hochberg method (7).

References

1. Sampaziotis F, Justin AW, Tysoe OC, Sawiak S, Godfrey EM, Upponi SS, et al. Reconstruction of the mouse extrahepatic biliary tree using primary human extrahepatic cholangiocyte organoids. Nat. Med. 2017;23:954–963.
2. Kraiczy J, Nayak KM, Howell KJ, Ross A, Forbester J, Salvestrini C, et al. DNA methylation defines regional identity of human intestinal epithelial organoids and undergoes dynamic changes during development. Gut. 2017;0:1–13.
3. Vallier L, Alexander M, Pedersen RA. Activin/Nodal and FGF pathways cooperate to maintain pluripotency of human embryonic stem cells. J. Cell Sci. 2005;118:4495–4509.
4. Patro R, Duggal G, Love MI, Irizarry RA, Kingsford C. Salmon provides fast and bias-aware quantification of transcript expression. Nat. Methods. 2017;14:417–419.
5. Soneson C, Love MI, Robinson MD. Differential analyses for RNA-seq: transcript-level estimates improve gene-level inferences. F1000Research. 2016;4:1521.
6. Love MI, Huber W, Anders S. Moderated estimation of fold change and dispersion for RNA-seq data with DESeq2. Genome Biol. 2014;15:550.
7. **Eden E, Navon R,** Steinfeld I, Lipson D, Yakhini Z. GOrilla: a tool for discovery and visualization of enriched GO terms in ranked gene lists. BMC Bioinformatics. 2009;10:48.

*Co-first authors are indicated in **bold***

<u>Supplementary Table Legends</u>

Supplementary Table 1. Donor Demographics

Supplementary Table 2. Reagents used for cell culture

Supplementary Table 3. List of antibodies used

Supplementary Table 4. List of primers used for qPCR

Supplementary Table 5. List of screening compounds tested to assess differentiation capacity of EHBD organoids.

Supplementary Figure Legends

Supplementary Figure 1. Flow cytometry for KRT19 and KRT7 in extrahepatic bile duct organoid cell lines.

Flow cytometry plots for KRT19 and KRT7 co-stained cells from individual GBD (n=1 donor) and CBD (n=4 donors) organoid cell lines.

Supplementary Figure 2. qPCR characterization of extrahepatic bile duct organoids over time in culture demonstrates maintanence of biliary and adult stem cell markers.

qPCR analyses showing the expression of biliary and adult stem cell markers in extrahepatic bile duct organoids (n=3-11, n=2-7 donor lines per passage) derived from common bile duct (CBD), gallbladder (GBD), and pancreatic duct (PancD) tissues over time in culture (P0, P5, P10 or higher). Gene expression is normalized to the housekeeping gene UBC and data is plotted as mean and SEM. *= $p \leq 0.05$, **= $p \leq 0.01$, ***= $p \leq 0.001$, **** $p \leq 0.0001$.

Supplementary Figure 3. Immunofluorescence images of extrahepatic bile duct organoids.

Single channel fluorescent images of extrahepatic bile duct organoids stained for various markers.

Supplementary Figure 4. Immunofluorescence images of extrahepatic biliary tissues.

Single channel fluorescent images of extrahepatic biliary tissues stained for various markers.

Supplementary Figure 5. Gene ontology analyses of extrahepatic biliary tissues.

(A) Top ten most significant gene ontologies (biological process) for 1,665 genes upregulated in gallbladder (GBD) compared to pancreatic duct (PancD) tissue. (B) Top ten most significant gene ontologies (biological process) for 765 genes upregulated in GBD compared to common bile duct (CBD) tissue. (C) Top ten most significant gene ontologies (biological process) for 1437 genes upregulated in PancD compared to GBD tissue. (D) Top ten most significant gene ontologies (biological process) for 1831 genes upregulated in PancD compared to CBD tissue. (E) Top ten

most significant gene ontology terms (biological process) for 716 genes upregulated in CBD compared to GBD tissue. (F) Top ten most significant gene ontology terms (biological process) for 1884 genes upregulated in CBD compared to PancD tissue. (G) Venn diagram showing the number, as well as overlap, of genes upregulated in either GBD compared to PancD tissue or CBD compared to PancD tissue. (H) Top ten most significant gene ontology terms (biological process) for 856 genes upregulated in both GBD and CBD tissues compared to PancD tissue.

Supplementary Figure 6. RNA-Sequencing analysis assessing for upregulation of progenitor markers in extrahepatic bile duct organoids compared to primary tissues.

(A-D) Genes upregulated in EHBD organoids compared to their tissue of origin which overlap with the intestinal stem cell signature described by Munoz et al (2012). 493 genes described by Munoz et al (2012) had human orthologs and were included in these analyses. (A) Venn diagrams showing the number of genes upregulated in EHBD organoids compared to their tissue of origin and the overlap of these genes with the intestinal stem cell signature. (B) Venn diagram showing the overlap of genes upregulated in CBD, GBD, or PancD organoids compared to their tissue of origin that were found to overlap with intestinal stem cell signature genes. (C) Heatmap of the variance stabilized counts of 135 intestinal stem cell signature genes that were upregulated in at least two out of the three EHBD organoid types compared to their tissue of origin. (D) Top twenty gene ontology terms enriched in the 135 intestinal stem cell signature genes which were upregulated in at least two out of the three EHBD organoid types compared to their tissue of origin. (E) Selected plots of RNA-Seq normalized counts of WNT pathway, pluripotency, and Non-WNT progenitor cell markers significantly upregulated in EHBD organoids compared to their tissue of origin.

Supplementary Figure 7. Gene ontology analyses of extrahepatic tissues and organoids.

(A) Top ten most significant gene ontologies (biological process) for the top 200 differentially expressed genes upregulated in CBD, GBD, or PancD tissues compared to their respective organoids. (B) Top ten most significant gene ontologies (biological process) for the top 200 differentially expressed genes upregulated in CBD, GBD, or PancD organoids compared to their respective tissue of origin. Significance cutoff: abs (log_2Fold Change) > 1 and smallest adj. p-values.

Supplementary Figure 8. Immunofluorescence images of extrahepatic bile duct organoids and extrahepatic tissue for regional specific markers SOX17 and CDX2.

Single channel fluorescent images of EHBD organoids and tissues for SOX17 and CDX2.

Supplementary Figure 9. Expression of SOX17, CDX2, and HOXB2 over time in culture in extrahepatic bile duct organoids.

qPCR analyses showing the expression of SOX17, CDX2, and HOXB2 in extrahepatic bile duct organoids (n=3-7, n=2-7 donor lines per passage) derived from common bile duct (CBD), gallbladder (GBD), or pancreatic duct (PancD) tissues over time in culture (P0, P5, P10 or higher). Gene expression is normalized to the housekeeping gene UBC and data is plotted as mean and SEM.

Supplementary Figure 10. Characterization of intrahepatic bile duct organoids isolated and cultured in extrahepatic conditions and the effects of CHIR 99021 on intrahepatic organoids.

(A) Images of intrahepatic bile duct organoids demonstrating the morphological heterogeneity of organoids cultured in extrahepatic media conditions (IHBD_CHIR) with both cystic and cryptic/budding organoids in the cultures. Scale bar = 1000 or 400 µm. (B) Immunofluorescence of IHBD_CHIR organoids for KI67 and ß-Catenin. White arrows indicate cystic organoids. Scale bar = 100 µm. (C) Number of organoids formed per 1,000 viable single cells plated from either GBD, CBD, or PancD organoids that were cultured for seven days in either extrahepatic media containing CHIR 99021 (EHBD ctrl), extrahepatic media without A8301 or FSK (-A8301/FSK), extrahepatic media without CHIR 99201 (-CHIR), extrahepatic media with 100ng/mL of DKK1 added (+DKK1), or extrahepatic media with 2.5uM IWP2 added (+IWP2). Data is plotted as mean and SEM and normalized relative to EHBD ctrl conditions (n=3 independent experiments, n=1 donor cell line per region; one-way ANOVA). *=$p \leq 0.05$.

Supplementary Figure 11. Immunofluorescence images of intrahepatic bile duct organoids and liver tissue.

Single channel fluorescent images of IHBD organoids and liver tissue.

Supplementary Figure 12. Expression profile of IHBD_NO CHIR organoids cultured in Intrahepatic Huch et al (2015) media or Extrahepatic media, with or without the addition of CHIR 99021.

qPCR analysis of IHBD_NO CHIR organoids (n=3 donor lines) transferred into various media conditions to assess the effect of CHIR 99021 on the expression profile of the organoids. Conditions included Extrahepatic organoid media with CHIR 99021 (n=6) or without CHIR 99021 (n=6) and Huch et al (2015) conditions with CHIR 99021 (n=7) or without CHIR (n=6). # = $p \leq 0.05$, ## = $p \leq 0.01$, ### = $p \leq 0.001$, #### = $p \leq 0.0001$ for the particular media condition compared to Huch et al Conditions without CHIR. If not otherwise indicated, comparisons between groups were not significantly different ($p>0.05$)

Supplementary Figure 13. Differentiation of IHBD_CHIR organoids

(A) qPCR analyses for stem cell, biliary, and liver genes in IHBD_CHIR organoids (n=2 independent experiments, n=1 donor line) in either expansion media (EM) or differentiation media (DM). Primary human hepatocytes (n=3) were used as positive controls. Gene expression is normalized to the housekeeping gene UBC and data is plotted as mean and SEM. (B) Immunofluorescence analysis for Albumin of IHBD_CHIR in either EM or DM conditions. Scale bar = 100 μm.

Supplementary Figure 14. Differentiation screening experiments on extrahepatic bile duct organoids to assess for hepatocyte differentiation ability.

(A) Diagram depicting the experimental design and timing. (B) qPCR analyses of extrahepatic bile duct organoids (n=3-7 donor cell lines per condition, except for TTR where A8301 and FSK conditions only have n=2) treated with a single screening factor for 4-6 days. H9 embryonic stem cells (H9, n=3) and primary hepatocytes (PH, n=3) were used as controls. Gene expression is normalized to the housekeeping gene, UBC, and data is plotted as mean and SEM. EM = extrahepatic bile duct expansion media. BM = basal media alone. # = $p \leq 0.05$, ## = $p \leq 0.01$ for the screening condition compared to EM. (C) Images of organoids treated with each of the screening conditions. Scale bar = 1000 μm.

Tissue	Donor Demographics	Tissue Sample / Cell Line	RNA-Sequencing T=tissue O=organoids
**Gallbladder**	21, Male	Tissue and Cell Line (-A8301/FSK)	Not sequenced
**Gallbladder**	33, Female	Tissue and Cell Line (-A8301/FSK)	Not sequenced
**Gallbladder**	39, Male	Tissue and Cell Line	Not sequenced
**Gallbladder**	21, Female	Tissue and Cell Line	T
**Gallbladder**	55, Female	Tissue and Cell Line	Not sequenced
**Gallbladder**	44, Male	Tissue and Cell Line	T
**Gallbladder**	63, Male	Tissue and Cell Line	T/O
**Gallbladder**	61, Female	Tissue and Cell Line	T/O
**Gallbladder**	67, Male	Tissue and Cell Line	T/O
**Gallbladder**	67, Male	Tissue and Cell Line	Not sequenced
**Gallbladder**	68, Female	Cell Line Only	Not sequenced
**Common Bile Duct**	50, Female	Cell Line only (-A8301/FSK)	Not Sequenced
**Common Bile Duct**	63, Male	Tissue and Cell Line	T/O
**Common Bile Duct**	20, Male	Tissue and Cell Line	T/O
**Common Bile Duct**	67, Male	Cell Line Only	O
**Common Bile Duct**	58, Female	Tissue Only	T
**Common Bile Duct**	43, Female	Tissue Only	T
**Common Bile Duct**	48, Female	Tissue and Cell Line	T/O
**Common Bile Duct**	36, Male	Tissue and Cell Line	T/O
**Common Bile Duct**	48, Female	Tissue and Cell Line	T/O
**Common Bile Duct**	61, Female	Cell Line Only	Not sequenced
**Pancreatic Duct**	42, Male	Tissue and Cell Line	T/O
**Pancreatic Duct**	27, Female	Tissue and Cell Line	T/O
**Pancreatic Duct**	37, Male	Cell Line Only	O
**Pancreatic Duct**	53 , Male	Tissue Only	T
**Liver**	50, Male	Cell Line Only (IHBD_CHIR, IHBD_NO CHIR)	O- IHBD_CHIR O- IHBD_NO CHIR
**Liver**	30, Female	Cell Line Only (IHBD_CHIR)	O- IHBD_CHIR
**Liver**	50, Female	Cell Line Only (IHBD_CHIR)	O- IHBD_CHIR
**Liver**	67, Female	Cell Line (IHBD_CHIR, IHBD_NO CHIR)	O- IHBD_NO CHIR
**Liver**	76, Male	Cell Line (IHBD_CHIR, IHBD_NO CHIR)	O- IHBD_NO CHIR

**Basal Organoid Medium = ADF+**	**Final Concentration**	**Supplier (catalog number)**
Advanced DMEM/F12 (ADF)	N/A	Thermo Fisher (12634010)
N2 Supplement 100X	1 X	Thermo Fisher (17502048)
B27™ Supplement 50X, serum-free	1 X	Thermo Fisher (17504044)
L-Glutamine	2 mM	Thermo Fisher (25030081)
Penicillin/Streptomycin	100 U/mL / 100 µg/mL	Thermo Fisher (15140122)
**Extrahepatic Organoid Expansion Media** (EHBD organoids or IHBD_CHIR organoids)	**Final Concentration**	**Supplier (catalog number)**
ADF+	See above	See above
RSPO-conditioned media<br><br><u>or</u><br><br>Recombinant Human R-Spondin 1	20% (vol/vol)<br><br><br><br>500 ng/mL	Cambridge Stem Cell Institute (homemade)<br><br><br>R&D (4645-RS)
CHIR 99021	3 µM	Tocris (4423)
Recombinant Human Noggin Fc Chimera	100 ng/mL	R&D (3344-NG)
Prostaglandin E_2	2.5 µM	R&D (2296)
Recombinant Human EGF, carrier free	100 ng/mL	R&D (236-EG)
A 83-01	5 µM	Tocris (2939)
Forskolin (FSK)	10 µM	Sigma (F6886)
Rock Inhibitor Y-27632<br>(added for first two days after isolation or splitting)	10 µM	Selleck Chem (S1049)
**Intrahepatic Organoid Isolation Media** (IHBD_NO CHIR organoids for first 3 days after isolation)	**Final Concentration**	**Supplier (catalog number)**
ADF+	See above	See above
RSPO-conditioned media	10% vol/vol	Cambridge Stem Cell Institute (homemade)
Wnt-conditioned media	30% vol/vol	Cambridge Stem Cell Institute (homemade)
Recombinant Human Noggin Fc Chimera	25 ng/mL	R&D (3344-NG)
N-acetylcysteine	1.25 mM	Sigma (A9165)
Nicotinamide	10 mM	Sigma (N0636)
Gastrin	10 nM	Sigma (G9145) or R&D (3006/1)
Recombinant Human EGF, carrier free	50 ng/mL	R&D (236-EG)
Recombinant Human HGF	25 ng/mL	Peprotech (100-39)
Recombinant Human FGF-10	100 ng/mL	Peprotech (100-26) or Autogen BioClear (ABC144)
A 83-01	5 µM	Tocris (2939)
Forskolin (FSK)	10 µM	Sigma (F6886)
Rock Inhibitor Y-27632	10 µM	Selleck Chem (S1049)

**Intrahepatic Organoid Expansion Media** (IHBD_NO CHIR organoids)	Final Concentration	Supplier (catalog number)
ADF+	See above	See above
RSPO-conditioned media	10% vol/vol	Cambridge Stem Cell Institute (homemade)
N-acetylcysteine	1.25 mM	Sigma (A9165)
Nicotinamide	10 mM	Sigma (N0636)
Human Gastrin I	10 nM	Sigma (G9145) or R&D (3006/1)
Recombinant Human EGF, carrier free	50 ng/mL	R&D (236-EG)
Recombinant Human HGF	25 ng/mL	Peprotech (100-39)
Recombinant Human FGF-10	100 ng/mL	Peprotech (100-26) or Autogen BioClear (ABC144)
A 83-01	5 μM	Tocris (2939)
Forskolin (FSK)	10 μM	Sigma (F6886)
Rock Inhibitor Y-27632 (added only for first two days after splitting)	10 μM	Selleck Chem (S1049)
**Huch et al (2015) Differentiation Media Phase I** (for Figure 8 experiments)	**Final Concentration**	**Supplier (catalog number)**
Intrahepatic Organoid Expansion Media	See above	See above
Recombinant Human BMP-7	25ng/mL	Peprotech (120-03)
**Huch et al (2015) Differentiation Media Phase II** (for Figure 8 experiments)	**Final Concentration**	**Supplier (catalog number)**
ADF+	See above	See above
N-acetylcysteine	1.25 mM	Sigma (A9165)
Gastrin	10 nM	Sigma (G9145) or R&D (3006/1)
Recombinant Human EGF, carrier free	50 ng/mL	R&D (236-EG)
Recombinant Human HGF	25 ng/mL	Peprotech (100-39)
A 83-01	0.5 μM	Tocris (2939)
Recombinant Human BMP-7	25ng/mL	Peprotech (120-03)
Dexamethasone	3 μM	Sigma (D4902)
DAPT	10 μM	Sigma (D5942)
Recombinant Human FGF19, carrier free	100 ng/mL	R&D (969-FG/CF)
**Additional Reagents used for cell culture and liver tissue digestion**	**Final Concentration**	**Supplier (catalog number)**
Cell Banker 2	N/A	Amsbio (11891)
Growth Factor Reduced Matrigel (with or without phenol red)	100%	Corning (354230) or (356231)
Cell Recovery Solution	N/A	Corning (354253)
TrpLE Express	N/A	Thermo Fisher (12604013)
Dispase II	128.4 μg/mL	Gibco 17105-041
Collagenase from *Clostridium histolyticum*	128.4 μg/mL	Sigma C9407
DMEM High Glucose + Glutamax	N/A	Thermo Fisher (10566016)
HBSS	N/A	Thermo Fisher (14175095)

Antibody	Species	Company/Product #	Dilution	Application
**EPCAM**	Mouse Monoclonal	R&D / MAB9601	1:100	IC<br>IC-Fr
**KRT7**	Rabbit Monoclonal	AbCam / ab68459	1:100	IC<br>IC-Fr<br>FC
**KRT7**	Mouse Monoclonal	AbCam / ab9021	1:100	IC<br>IC-Fr
**KRT19**	Mouse Monoclonal	AbCam / ab7754	1:100	IC<br>IC-Fr<br>FC
**HNF4α**	Rabbit Monoclonal	AbCam / ab92378	1:100	IC<br>IC-Fr
**SOX9**	Rabbit Monoclonal	AbCam / ab185230	1:100	IC<br>IC-Fr
**β-Catenin**	Goat Polyclonal	R&D / AF1329	1:100	IC<br>IC-Fr
**KI67**	Rabbit Monoclonal	AbCam / ab15580	1:100	IC<br>IC-Fr
**CDX2**	Rabbit Monoclonal	AbCam / ab76541	1:100	IC<br>IC-Fr
**SOX17**	Goat Polyclonal	R&D / AF1924	1:100	IC<br>IC-Fr
**Alexa Fluor 488**	Donkey anti-goat IgG	Invitrogen / A11055	1:1000	IC<br>IC-Fr
**Alexa Fluor 488**	Donkey anti-mouse IgG	Invitrogen / A10037	1:1000	IC<br>IC-Fr<br>FC
**Alexa Fluor 488**	Chicken anti-mouse IgG	Thermo / A21200	1:1000	IC<br>IC-Fr
**Alexa Fluor 488**	Chicken anti-goat IgG	Thermo / A21467	1:1000	IC<br>IC-Fr
**Alexa Fluor 568**	Donkey anti-goat IgG	Invitrogen / A11057	1:1000	IC<br>IC-Fr
**Alexa Fluor 568**	Donkey anti-mouse IgG	Invitrogen / A10037	1:1000	IC<br>IC-Fr
**Alexa Fluor 568**	Donkey anti-rabbit IgG	Invitrogen / A10042	1:1000	IC<br>IC-Fr
**Alexa Fluor 647**	Donkey anti-rabbit IgG	Invitrogen / A31573	1:1000	FC
**TRITC**	Donkey anti-Goat IgG	Thermo / A16010	1:1000	IC<br>IC-Fr
**TRITC**	Donkey anti-rabbit IgG	Thermo / A16040	1:1000	IC<br>IC-Fr
**Hoechst 33258**	Nuclear Stain	Sigma / 94403	1:10,000	IC<br>IC-Fr

Gene	Forward Primer	Reverse Primer
**LGR5**	CTCCCAGGTCTGGTGTGTTG	GAGGTCTAGGTAGGAGGTGAAG
**PROM1**	AGTCGGAAACTGGCAGATAGC	GGTAGTGTTGTACTGGGCCAAT
**SOX9**	CTCTGGAGACTTCTGAACGAGAG	CCTTGAAGATGGCGTTGGGG
**HNF4A**	CATGGCCAAGATTGACAACCT	TTCCCATATGTTCCTGCATCAG
**ALBUMIN**	CCTTTGGCACAATGAAGTGGGTAACC	CAGCAGTCAGCCATTTCACCATAG
**TBX3**	TGGAGCCCGAAGAAGAGGTG	TTCGCCTTCCCGACTTGGTA
**CYP3A4**	TGTGCCTGAGAACACCAGAG	GTGGTGGAAATAGTCCCGTG
**TTR**	ATGGCTTCTCATCGTCTGCT	TGTCATCAGCAGCCTTTCTG
**KRT19**	ACGACCATCCAGGACCTGC	TCCCACTTGGCCCCTCAGC
**KRT7**	GATTGCTGGCCTTCGGGGT	TCATCACAGAGATATTCACGGCTC
**HNF1B**	GCACCCCTATGAAGACCCAG	GGACTGTCTGGTTGAATTGTCG
**CDX2**	GGCAGCCAAGTGAAAACCAG	TTCCTCTCCTTTGCTCTGCG
**SOX17**	CGCACGGAATTTGAACAGTA	GGATCAGGGACCTGTCACAC
**HOXB2**	CCTAGCCTACAGGGTTCTCTC	CACAGAGCGTACTGGTGAAAAA
**UBC**	ATTTGGGTCGCGGTTCTTG	TGCCTTGACATTCTCGATGGT

Compound	Pathway	Proliferation Impact	Hepatocyte Markers
Activin (50ng/mL)	TGFβ Activation	–	n.s.
SB 431542 (10μM)	TGFβ inhibition	-	n.s.
A 83-01 (5μM)	TGFβ inhibition	+	n.s.
BMP4 (25ng/mL)	BMP activation	-	n.s.
BMP7 (25ng/mL)	BMP activation	–	n.s.
Noggin (200ng/mL)	BMP inhibition	+	n.s.
Forskolin (10μM)	cAMP activation	+	n.s.
CHIR (3μM)	Wnt activation	- (alone)<br>+ (with RSPO)	n.s.
Wnt3a Cond. Media (30%)	Wnt activation	+/o	n.s.
DKK (100ug/ml)	Wnt inhibition	-	n.s.
DAPT (10μM)	Notch Inhibitior	–	n.s.
DBZ (10μM)	Notch Inhibition	-	n.s.
HGF (50-100ng/mL)	HGF signaling	+ (caused 2D growth in matrigel)	n.s.
Oncostatin-M (100ng/mL)	OSM signaling	+	n.s.
FGF10 (100ng/mL)	FGF signaling	+	n.s.
FGF7 (100ng/mL)	FGF signaling	+	n.s.
FGF2 (24ng/mL)	FGF signaling	–	n.s.
FGF19 (100ng/mL)	FGF signaling	o	n.s.
Dexamethasone (30 μM	GR signaling	–	n.s.
Hepatozyme Media	n/a	-	n.s.

GR= glucocorticoid receptor, + = increased, o = neither increased or decreased, - = decrease
n.s. = not significant

GBD Organoids

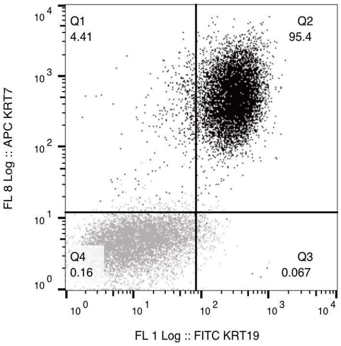

CBD Organoids

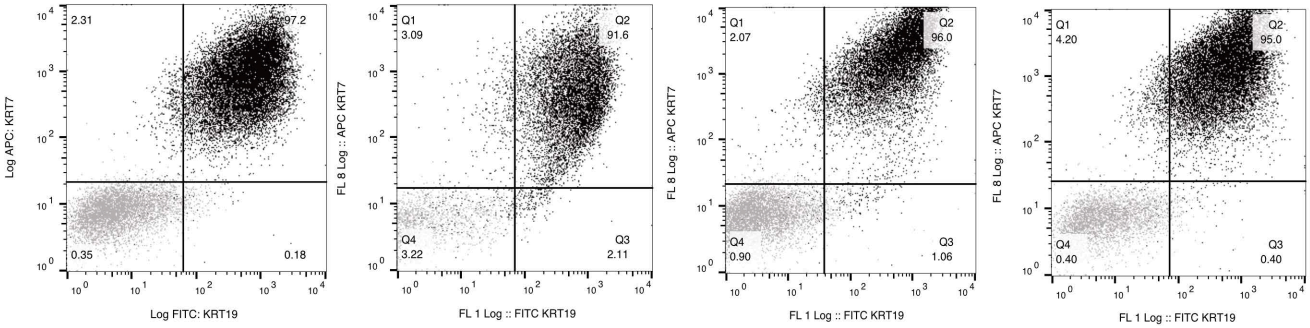

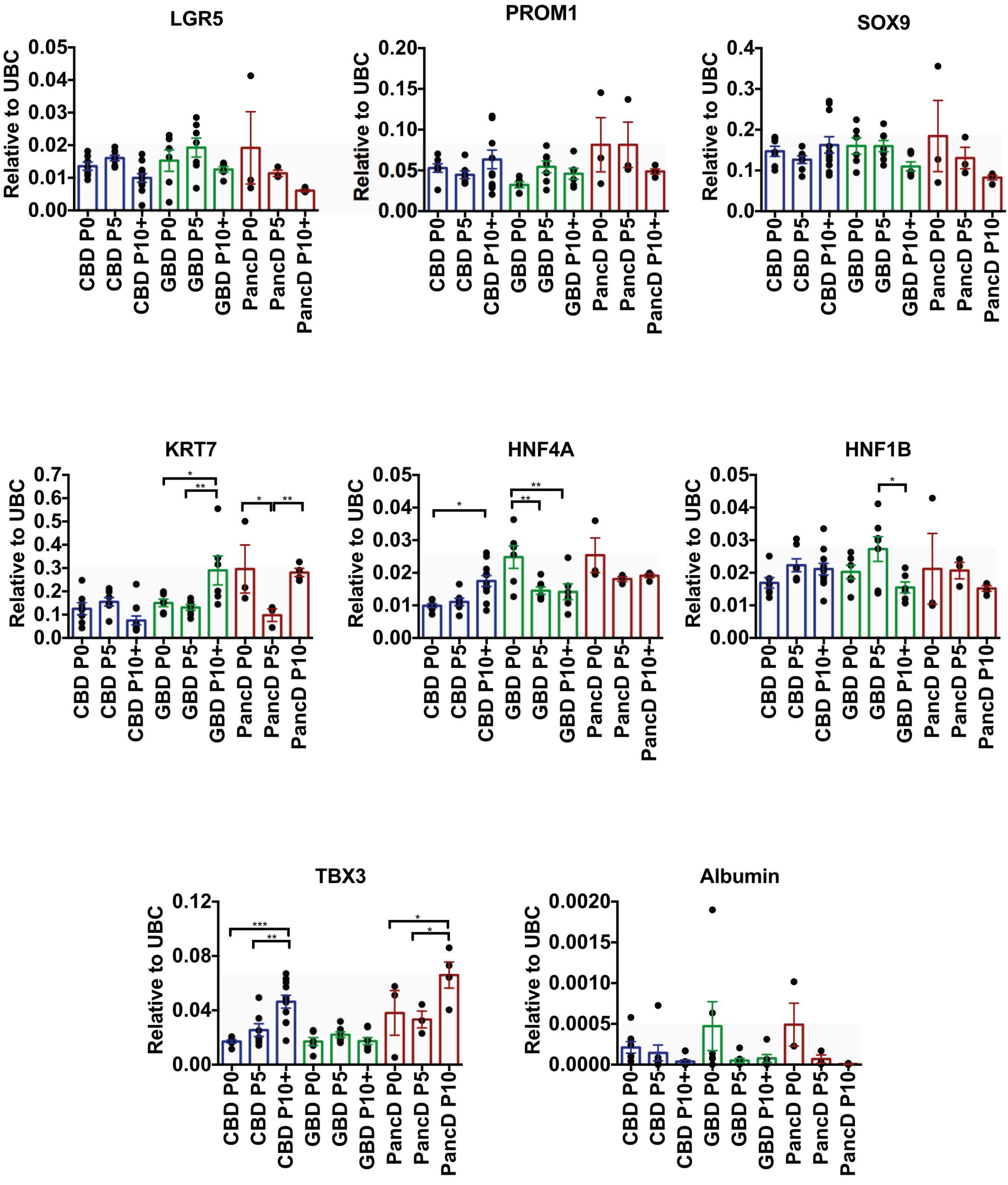
LGR5
PROM1
SOX9
KRT7
HNF4A
HNF1B
TBX3
Albumin
Relative to UBC
CBD P0
CBD P5
CBD P10+
GBD P0
GBD P5
GBD P10+
PancD P0
PancD P5
PancD P10+
PancD P10

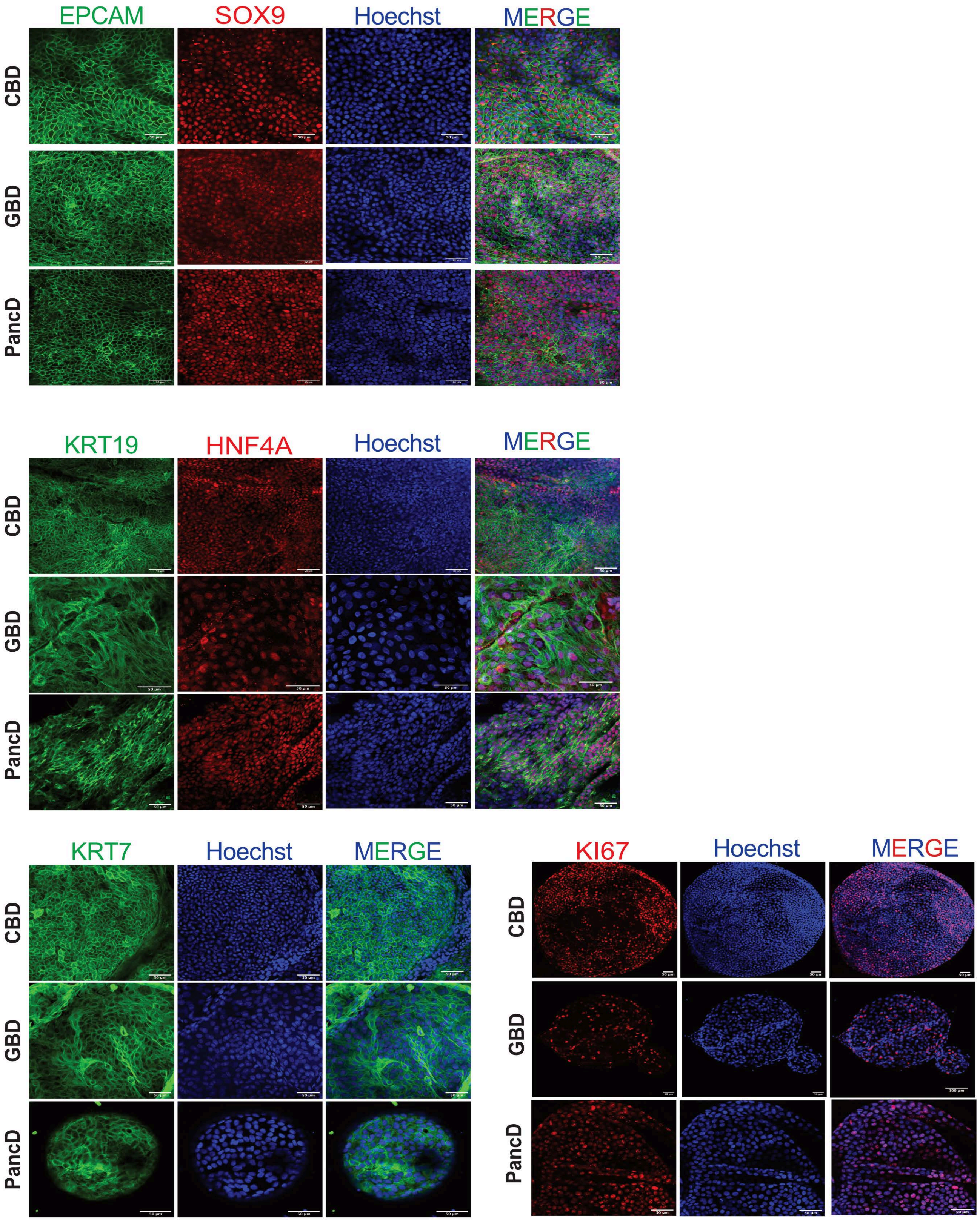
EPCAM
SOX9
Hoechst
MERGE
CBD
GBD
PancD
KRT19
HNF4A
Hoechst
MERGE
CBD
GBD
PancD
KRT7
Hoechst
MERGE
CBD
GBD
PancD
KI67
Hoechst
MERGE
CBD
GBD
PancD

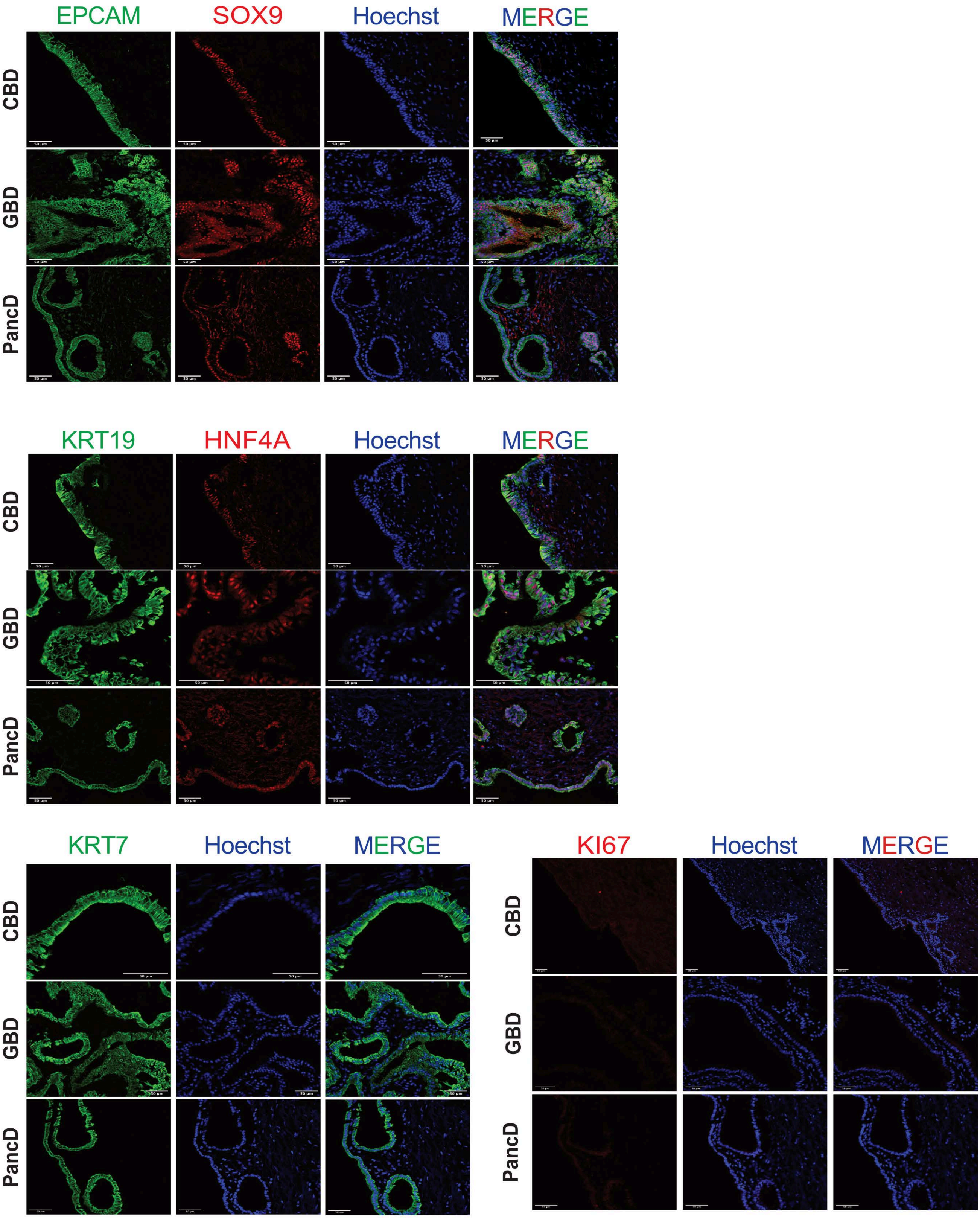
EPCAM
SOX9
Hoechst
MERGE
CBD
GBD
PancD
KRT19
HNF4A
Hoechst
MERGE
CBD
GBD
PancD
KRT7
Hoechst
MERGE
CBD
GBD
PancD
KI67
Hoechst
MERGE
CBD
GBD
PancD
50 µm

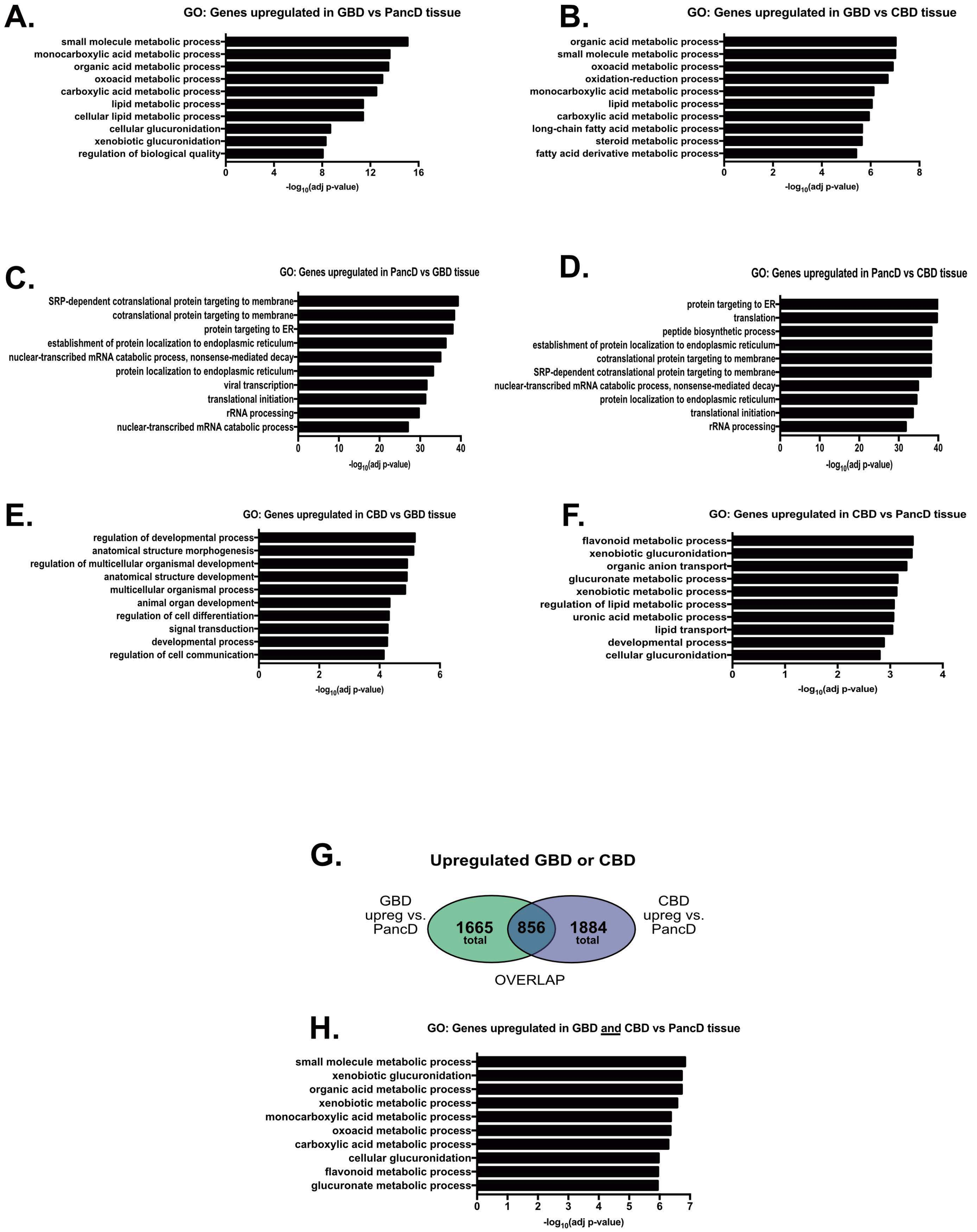

A.
GO: Genes upregulated in GBD vs PancD tissue
small molecule metabolic process
monocarboxylic acid metabolic process
organic acid metabolic process
oxoacid metabolic process
carboxylic acid metabolic process
lipid metabolic process
cellular lipid metabolic process
cellular glucuronidation
xenobiotic glucuronidation
regulation of biological quality
0 4 8 12 16
-log10(adj p-value)
B.
GO: Genes upregulated in GBD vs CBD tissue
organic acid metabolic process
small molecule metabolic process
oxoacid metabolic process
oxidation-reduction process
monocarboxylic acid metabolic process
lipid metabolic process
carboxylic acid metabolic process
long-chain fatty acid metabolic process
steroid metabolic process
fatty acid derivative metabolic process
0 2 4 6 8
-log10(adj p-value)
C.
GO: Genes upregulated in PancD vs GBD tissue
SRP-dependent cotranslational protein targeting to membrane
cotranslational protein targeting to membrane
protein targeting to ER
establishment of protein localization to endoplasmic reticulum
nuclear-transcribed mRNA catabolic process, nonsense-mediated decay
protein localization to endoplasmic reticulum
viral transcription
translational initiation
rRNA processing
nuclear-transcribed mRNA catabolic process
0 10 20 30 40
-log10(adj p-value)
D.
GO: Genes upregulated in PancD vs CBD tissue
protein targeting to ER
translation
peptide biosynthetic process
establishment of protein localization to endoplasmic reticulum
cotranslational protein targeting to membrane
SRP-dependent cotranslational protein targeting to membrane
nuclear-transcribed mRNA catabolic process, nonsense-mediated decay
protein localization to endoplasmic reticulum
translational initiation
rRNA processing
0 10 20 30 40
-log10(adj p-value)
E.
GO: Genes upregulated in CBD vs GBD tissue
regulation of developmental process
anatomical structure morphogenesis
regulation of multicellular organismal development
anatomical structure development
multicellular organismal process
animal organ development
regulation of cell differentiation
signal transduction
developmental process
regulation of cell communication
0 2 4 6
-log10(adj p-value)
F.
GO: Genes upregulated in CBD vs PancD tissue
flavonoid metabolic process
xenobiotic glucuronidation
organic anion transport
glucuronate metabolic process
xenobiotic metabolic process
regulation of lipid metabolic process
uronic acid metabolic process
lipid transport
developmental process
cellular glucuronidation
0 1 2 3 4
-log10(adj p-value)
G.
Upregulated GBD or CBD
GBD upreg vs. PancD
1665 total
856
1884 total
CBD upreg vs. PancD
OVERLAP
H.
GO: Genes upregulated in GBD and CBD vs PancD tissue
small molecule metabolic process
xenobiotic glucuronidation
organic acid metabolic process
xenobiotic metabolic process
monocarboxylic acid metabolic process
oxoacid metabolic process
carboxylic acid metabolic process
cellular glucuronidation
flavonoid metabolic process
glucuronate metabolic process
0 1 2 3 4 5 6 7
-log10(adj p-value)

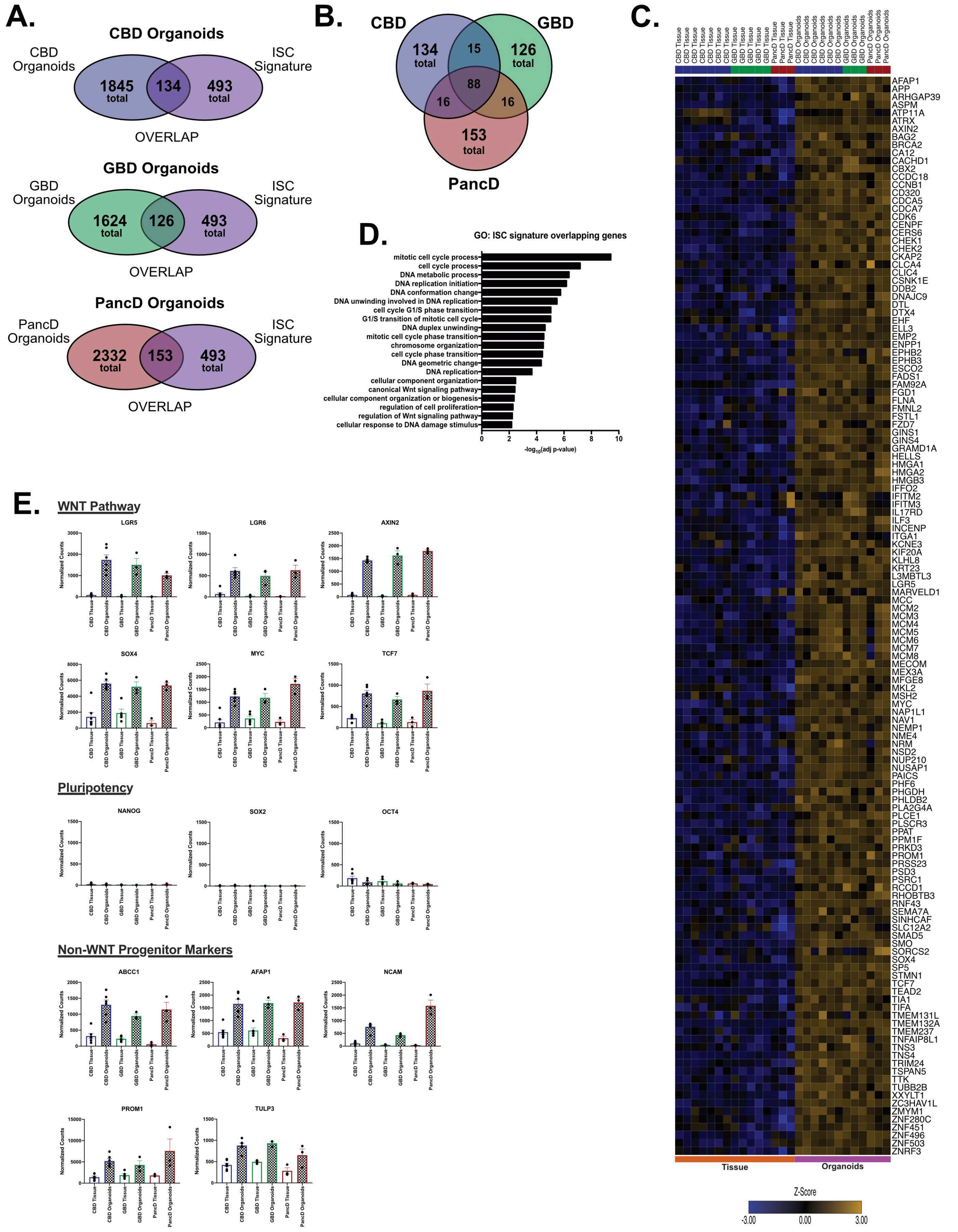
A.
CBD Organoids
CBD Organoids
ISC Signature
1845 total
134
493 total
OVERLAP
GBD Organoids
GBD Organoids
ISC Signature
1624 total
126
493 total
OVERLAP
PancD Organoids
PancD Organoids
ISC Signature
2332 total
153
493 total
OVERLAP
B.
CBD
GBD
134 total
15
126 total
88
16
16
153 total
PancD
C.
CBD Tissue
GBD Tissue
PancD Tissue
CBD Organoids
GBD Organoids
PancD Organoids
AFAP1
APP
ARHGAP39
ASPM
ATP11A
ATRX
AXIN2
BAG2
BRCA2
CA12
CACHD1
CBX2
CCDC18
CCNB1
CD320
CDCA5
CDCA7
CDK6
CENPF
CERS6
CHEK1
CHEK2
CKAP2
CLCA4
CLIC4
CSNK1E
DDB2
DNAJC9
DTL
DTX4
EHF
ELL3
EMP2
ENPP1
EPHB2
EPHB3
ESCO2
FADS1
FAM92A
FGD1
FLNA
FMNL2
FSTL1
FZD7
GINS1
GINS4
GRAMD1A
HELLS
HMGA1
HMGA2
HMGB3
IFFO2
IFITM2
IFITM3
IL17RD
ILF3
INCENP
ITGA1
KCNE3
KIF20A
KLHL8
KRT23
L3MBTL3
LGR5
MARVELD1
MCC
MCM2
MCM3
MCM4
MCM5
MCM6
MCM7
MCM8
MECOM
MEX3A
MFGE8
MKL2
MSH2
MYC
NAP1L1
NAV1
NEMP1
NME4
NRM
NSD2
NUP210
NUSAP1
PAICS
PHF6
PHGDH
PHLDB2
PLA2G4A
PLCE1
PLSCR3
PPAT
PPM1F
PRKD3
PROM1
PRSS23
PSD3
PSRC1
RCCD1
RHOBTB3
RNF43
SEMA7A
SINHCAF
SLC12A2
SMAD5
SMO
SORCS2
SOX4
SP5
STMN1
TCF7
TEAD2
TIA1
TIFA
TMEM131L
TMEM132A
TMEM237
TNFAIP8L1
TNS3
TNS4
TRIM24
TSPAN5
TTK
TUBB2B
XXYLT1
ZC3HAV1L
ZMYM1
ZNF280C
ZNF451
ZNF496
ZNF503
ZNRF3
Tissue
Organoids
Z-Score
-3.00
0.00
3.00
D.
GO: ISC signature overlapping genes
mitotic cell cycle process
cell cycle process
DNA metabolic process
DNA replication initiation
DNA conformation change
DNA unwinding involved in DNA replication
cell cycle G1/S phase transition
G1/S transition of mitotic cell cycle
DNA duplex unwinding
mitotic cell cycle phase transition
chromosome organization
cell cycle phase transition
DNA geometric change
DNA replication
cellular component organization
canonical Wnt signaling pathway
cellular component organization or biogenesis
regulation of cell proliferation
regulation of Wnt signaling pathway
cellular response to DNA damage stimulus
0 2 4 6 8 10
-log10(adj p-value)
E.
WNT Pathway
LGR5
LGR6
AXIN2
SOX4
MYC
TCF7
Pluripotency
NANOG
SOX2
OCT4
Non-WNT Progenitor Markers
ABCC1
AFAP1
NCAM
PROM1
TULP3
Normalized Counts
CBD Tissue
CBD Organoids
GBD Tissue
GBD Organoids
PancD Tissue
PancD Organoids

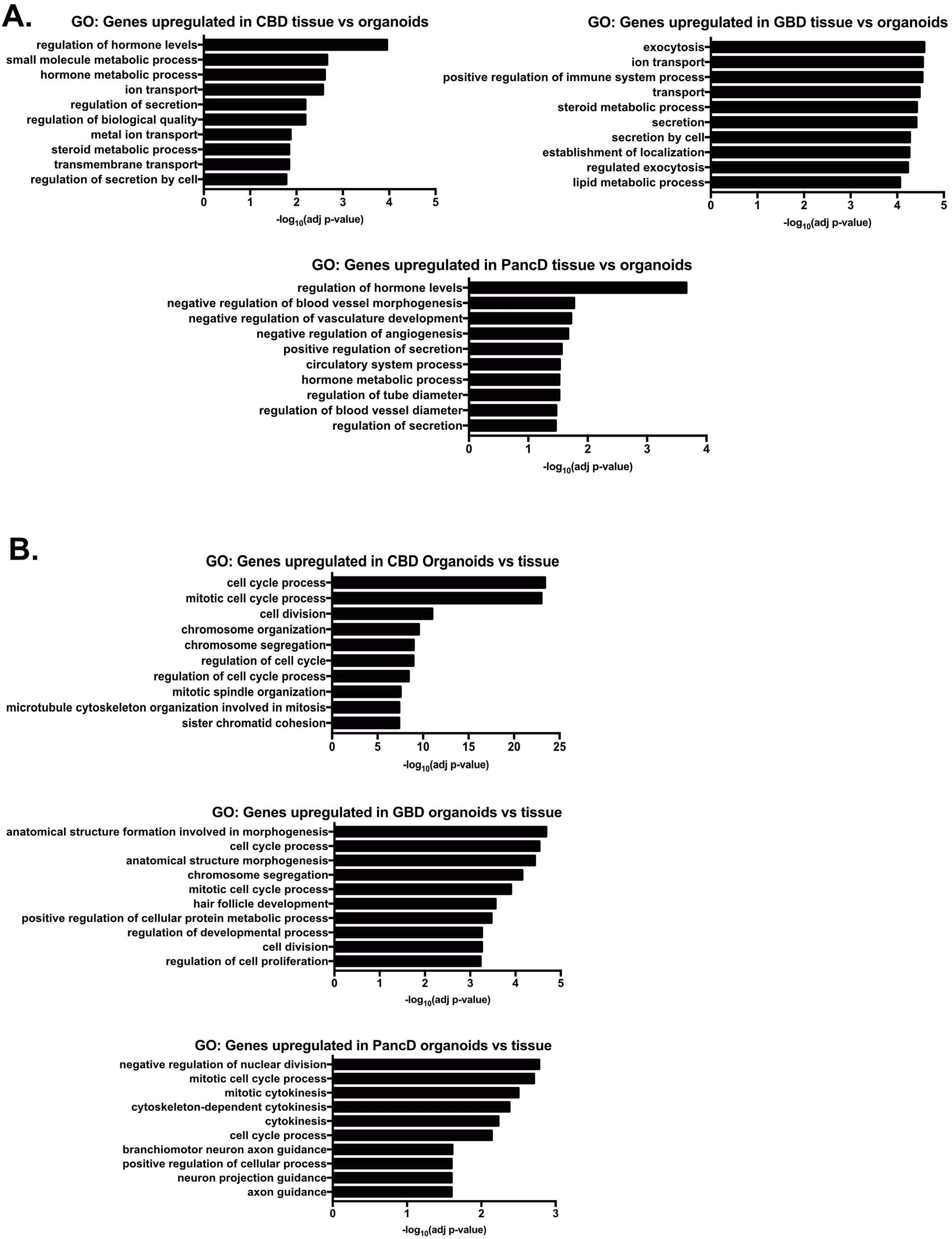
A.
GO: Genes upregulated in CBD tissue vs organoids
regulation of hormone levels
small molecule metabolic process
hormone metabolic process
ion transport
regulation of secretion
regulation of biological quality
metal ion transport
steroid metabolic process
transmembrane transport
regulation of secretion by cell
0 1 2 3 4 5
-log10(adj p-value)
GO: Genes upregulated in GBD tissue vs organoids
exocytosis
ion transport
positive regulation of immune system process
transport
steroid metabolic process
secretion
secretion by cell
establishment of localization
regulated exocytosis
lipid metabolic process
0 1 2 3 4 5
-log10(adj p-value)
GO: Genes upregulated in PancD tissue vs organoids
regulation of hormone levels
negative regulation of blood vessel morphogenesis
negative regulation of vasculature development
negative regulation of angiogenesis
positive regulation of secretion
circulatory system process
hormone metabolic process
regulation of tube diameter
regulation of blood vessel diameter
regulation of secretion
0 1 2 3 4
-log10(adj p-value)
B.
GO: Genes upregulated in CBD Organoids vs tissue
cell cycle process
mitotic cell cycle process
cell division
chromosome organization
chromosome segregation
regulation of cell cycle
regulation of cell cycle process
mitotic spindle organization
microtubule cytoskeleton organization involved in mitosis
sister chromatid cohesion
0 5 10 15 20 25
-log10(adj p-value)
GO: Genes upregulated in GBD organoids vs tissue
anatomical structure formation involved in morphogenesis
cell cycle process
anatomical structure morphogenesis
chromosome segregation
mitotic cell cycle process
hair follicle development
positive regulation of cellular protein metabolic process
regulation of developmental process
cell division
regulation of cell proliferation
0 1 2 3 4 5
-log10(adj p-value)
GO: Genes upregulated in PancD organoids vs tissue
negative regulation of nuclear division
mitotic cell cycle process
mitotic cytokinesis
cytoskeleton-dependent cytokinesis
cytokinesis
cell cycle process
branchiomotor neuron axon guidance
positive regulation of cellular process
neuron projection guidance
axon guidance
0 1 2 3
-log10(adj p-value)

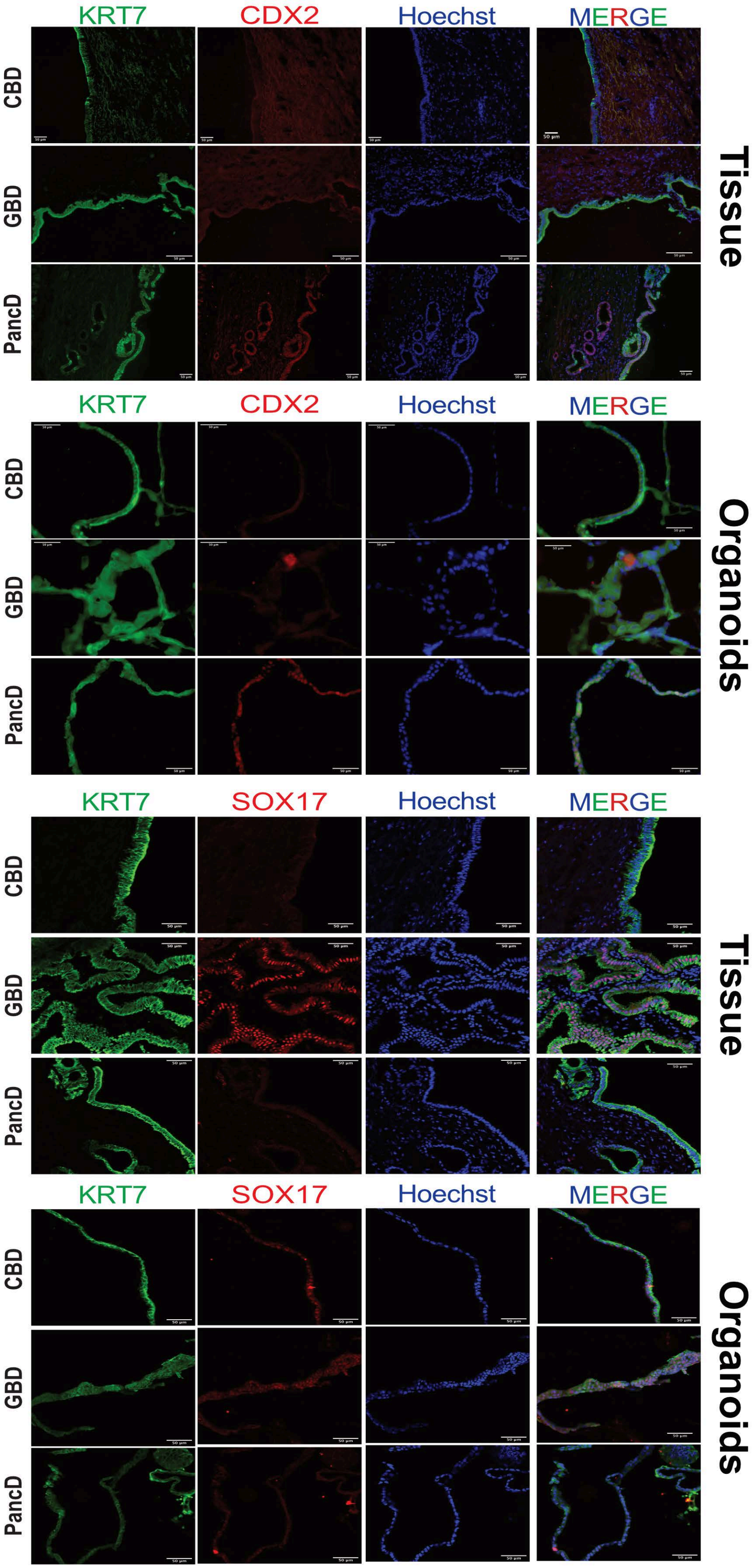

KRT7
CDX2
Hoechst
MERGE
CBD
GBD
PancD
Tissue
KRT7
CDX2
Hoechst
MERGE
CBD
GBD
PancD
Organoids
KRT7
SOX17
Hoechst
MERGE
CBD
GBD
PancD
Tissue
KRT7
SOX17
Hoechst
MERGE
CBD
GBD
PancD
Organoids

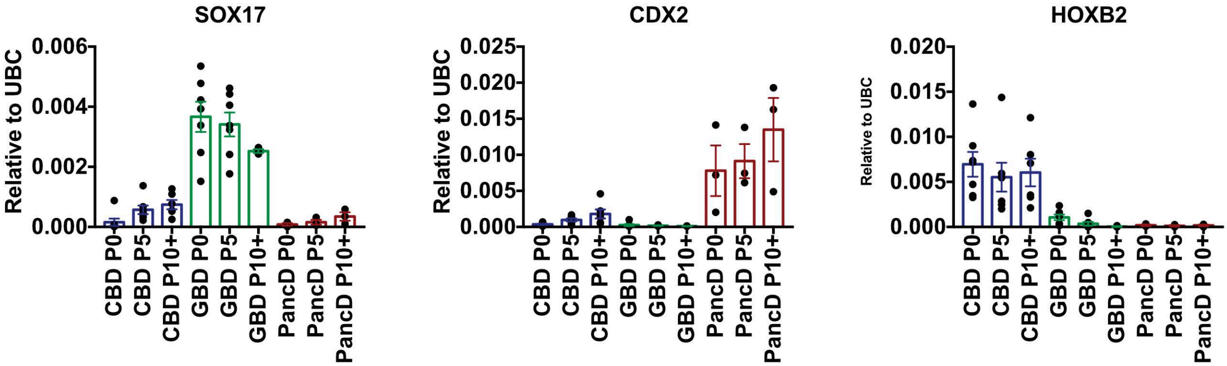
SOX17
CDX2
HOXB2
Relative to UBC
0.006
0.004
0.002
0.000
0.025
0.020
0.015
0.010
0.005
0.000
0.020
0.015
0.010
0.005
0.000
CBD P0
CBD P5
CBD P10+
GBD P0
GBD P5
GBD P10+
PancD P0
PancD P5
PancD P10+

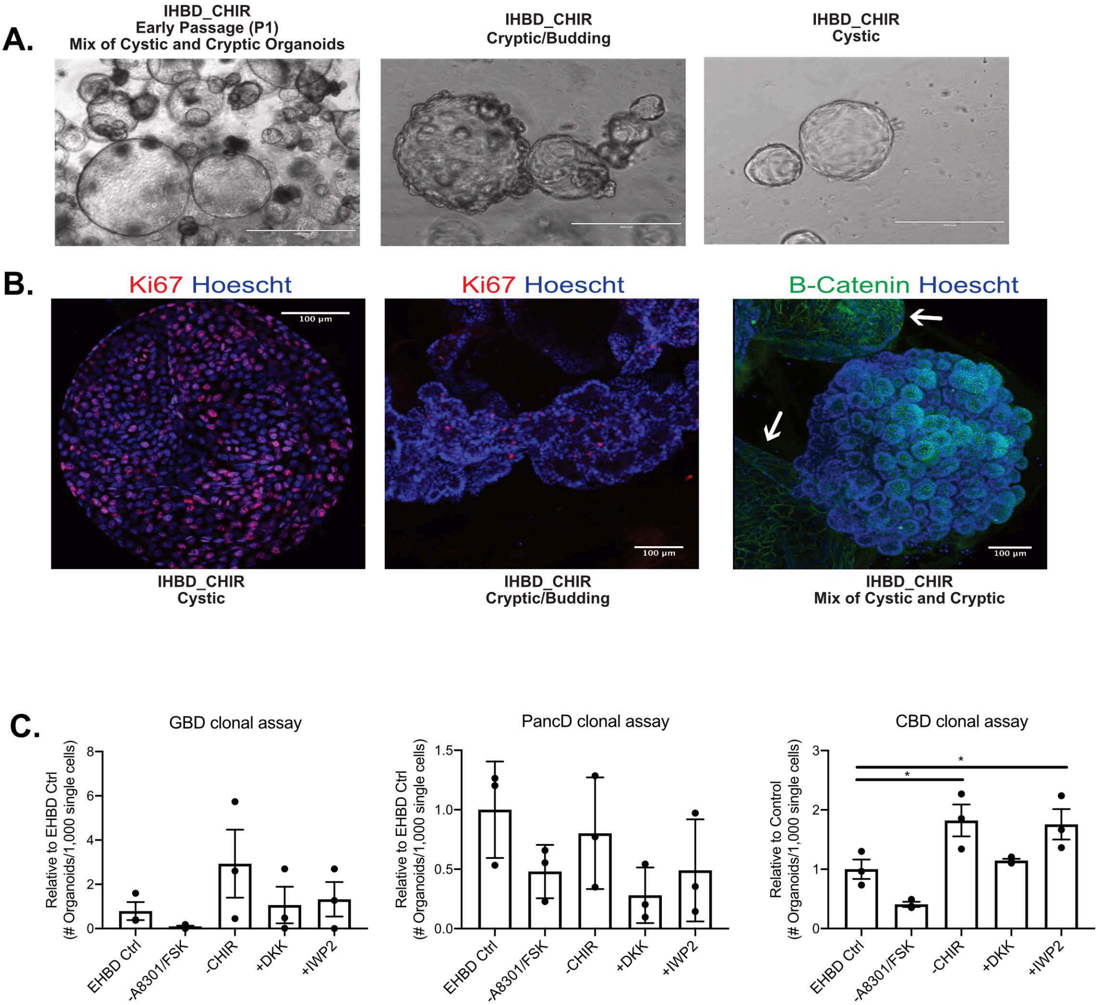
A.
IHBD_CHIR
Early Passage (P1)
Mix of Cystic and Cryptic Organoids
IHBD_CHIR
Cryptic/Budding
IHBD_CHIR
Cystic
B.
Ki67 Hoescht
Ki67 Hoescht
B-Catenin Hoescht
100 µm
100 µm
100 µm
IHBD_CHIR
Cystic
IHBD_CHIR
Cryptic/Budding
IHBD_CHIR
Mix of Cystic and Cryptic
C.
GBD clonal assay
Relative to EHBD Ctrl
(# Organoids/1,000 single cells)
PancD clonal assay
Relative to EHBD Ctrl
(# Organoids/1,000 single cells)
CBD clonal assay
Relative to Control
(# Organoids/1,000 single cells)
EHBD Ctrl
-A8301/FSK
-CHIR
+DKK
+IWP2
*
*

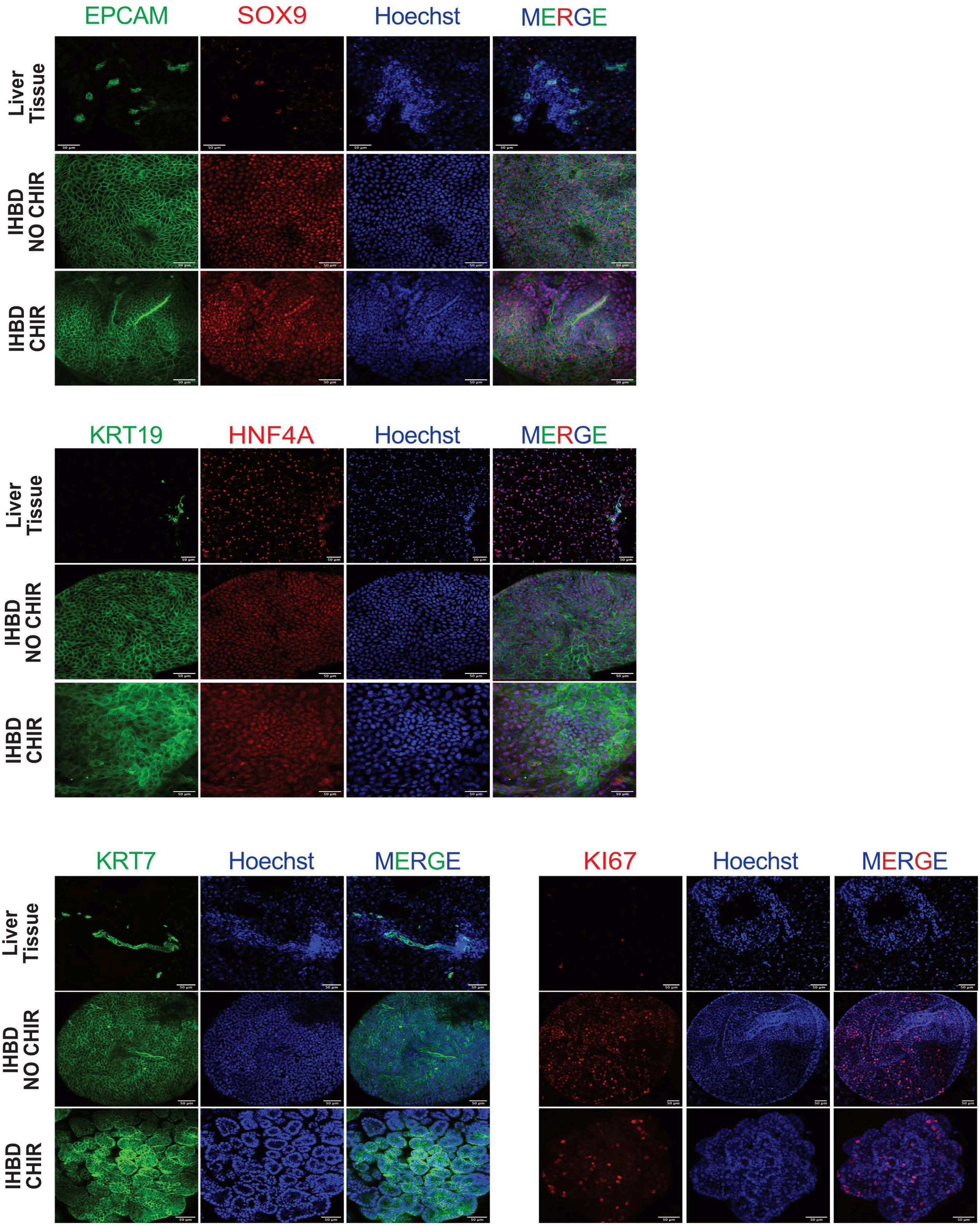
EPCAM
SOX9
Hoechst
MERGE
Liver Tissue
IHBD NO CHIR
IHBD CHIR
KRT19
HNF4A
Hoechst
MERGE
Liver Tissue
IHBD NO CHIR
IHBD CHIR
KRT7
Hoechst
MERGE
KI67
Hoechst
MERGE
Liver Tissue
IHBD NO CHIR
IHBD CHIR

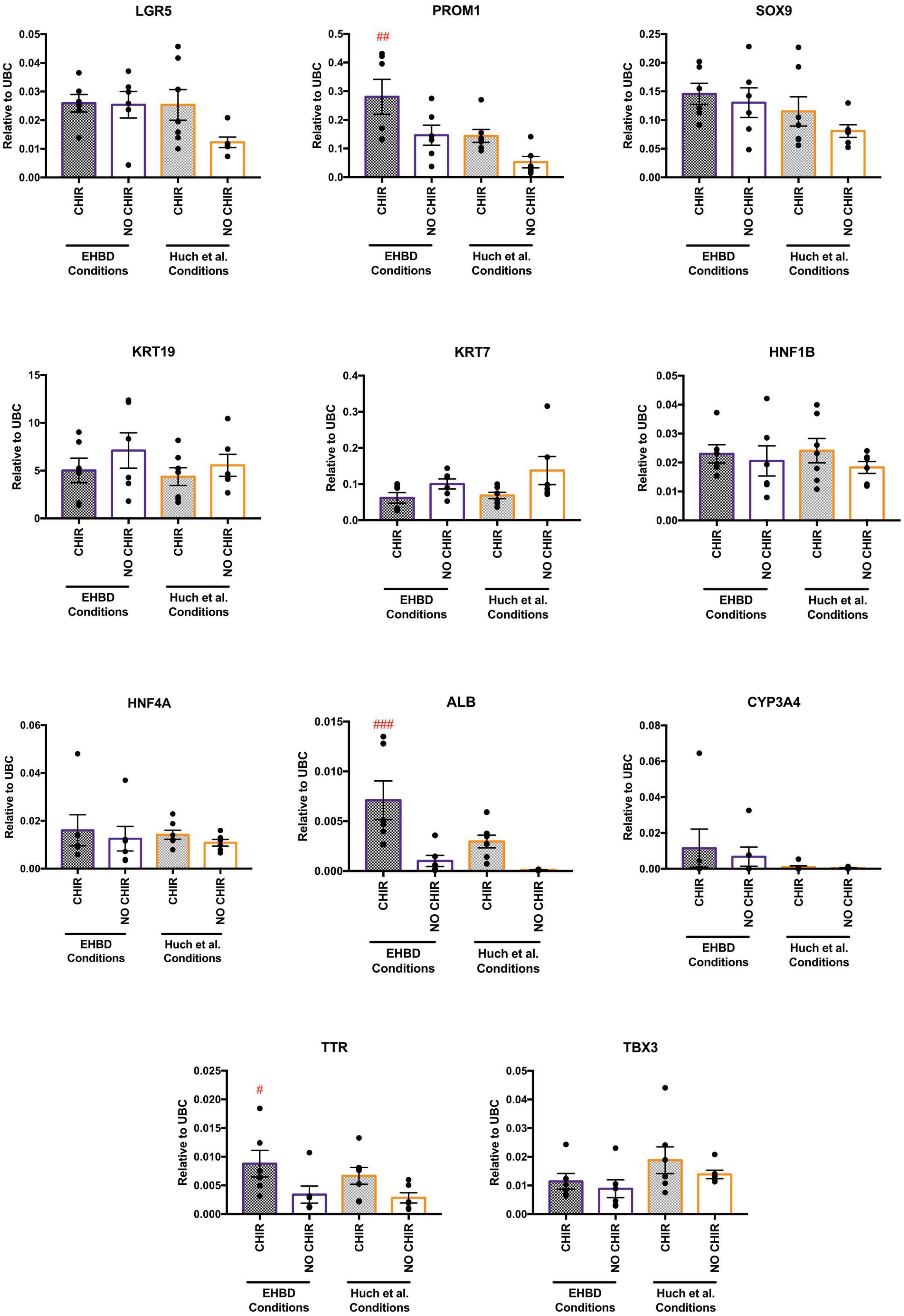

LGR5
Relative to UBC
0.00
0.01
0.02
0.03
0.04
0.05
CHIR
NO CHIR
CHIR
NO CHIR
EHBD Conditions
Huch et al. Conditions
PROM1
##
Relative to UBC
0.0
0.1
0.2
0.3
0.4
0.5
SOX9
Relative to UBC
0.00
0.05
0.10
0.15
0.20
0.25
KRT19
Relative to UBC
0
5
10
15
KRT7
Relative to UBC
0.0
0.1
0.2
0.3
0.4
HNF1B
Relative to UBC
0.00
0.01
0.02
0.03
0.04
0.05
HNF4A
Relative to UBC
0.00
0.02
0.04
0.06
ALB
###
Relative to UBC
0.000
0.005
0.010
0.015
CYP3A4
Relative to UBC
0.00
0.02
0.04
0.06
0.08
TTR
#
Relative to UBC
0.000
0.005
0.010
0.015
0.020
0.025
TBX3
Relative to UBC
0.00
0.01
0.02
0.03
0.04
0.05

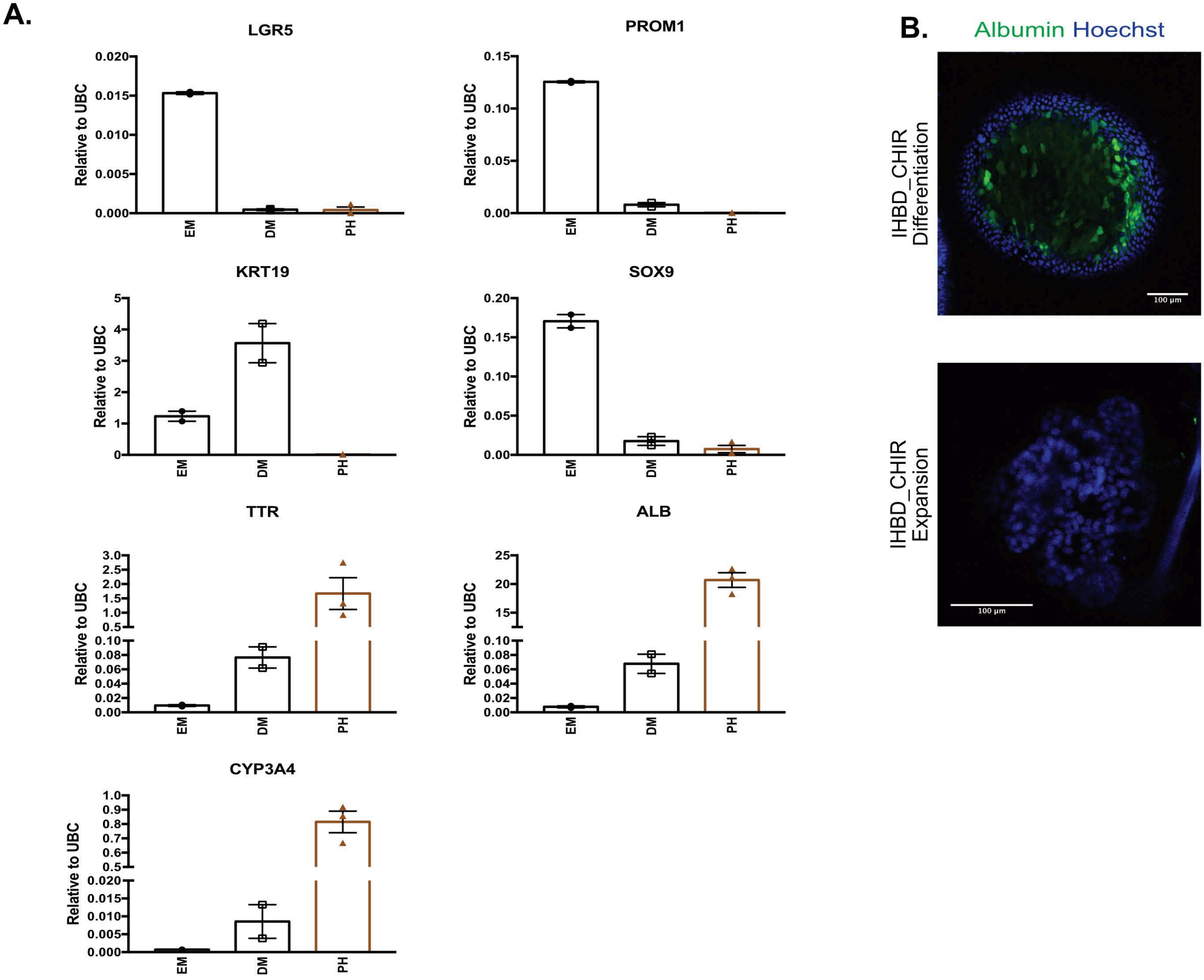
A.
LGR5
PROM1
KRT19
SOX9
TTR
ALB
CYP3A4
Relative to UBC
EM
DM
PH
B.
Albumin Hoechst
IHBD_CHIR Differentiation
IHBD_CHIR Expansion
100 µm

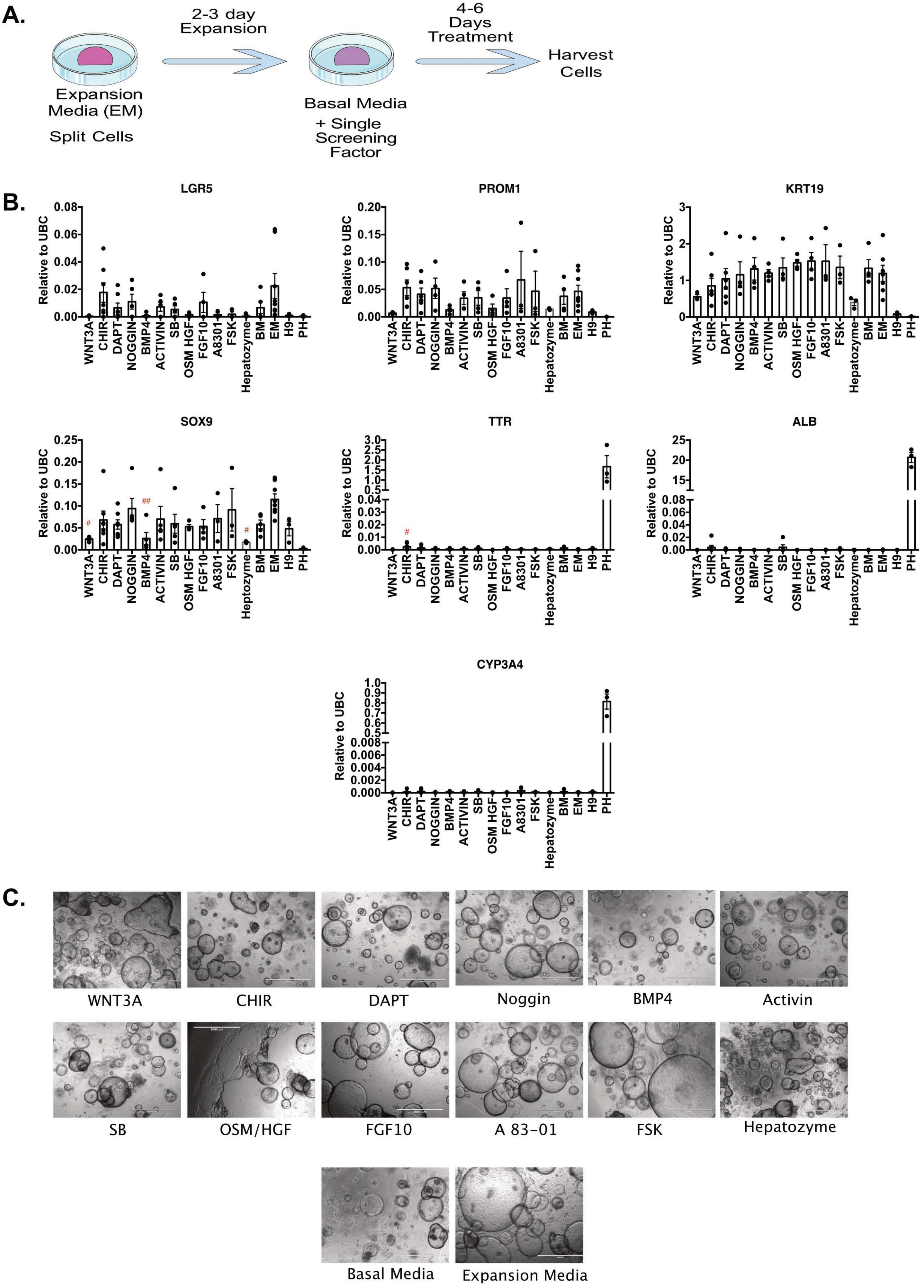
A.
2-3 day Expansion
4-6 Days Treatment
Expansion Media (EM)
Split Cells
Basal Media
+ Single Screening Factor
Harvest Cells
B.
LGR5
PROM1
KRT19
SOX9
TTR
ALB
CYP3A4
Relative to UBC
WNT3A
CHIR
DAPT
NOGGIN
BMP4
ACTIVIN
SB
OSM HGF
FGF10
A8301
FSK
Hepatozyme
Heptozyme
BM
EM
H9
PH
#
##
C.
WNT3A
CHIR
DAPT
Noggin
BMP4
Activin
SB
OSM/HGF
FGF10
A 83-01
FSK
Hepatozyme
Basal Media
Expansion Media
