# Supplementary material for: Regional Differences in Human Biliary Tissues and Corresponding In Vitro–Derived Organoids
Source: Hepatology. 2021 Feb 6;73(1):247–67. doi: 10.1002/hep.31252 (PMC8641381; doi:10.1002/hep.31252)
Supplement: Supplementary file 12 [file HEP-73-247-s008.docx]

**Supplementary Materials and Methods**

**Immunocytochemistry**

*Whole mount organoids*

Organoids were grown on 9 mm round glass coverslips (VWR) and then washed for 5 min in PBS, fixed in 4% paraformaldehyde (PFA) for 20 min at room temperature (RT), and blocked/permeabilized for 3 h in 10% donkey serum and 0.1% Triton-X. Coverslips were stained as previously described (1), except secondary antibodies were applied overnight at 4°C. Coverslips were removed using forceps and mounted on glass slides using Fluoromount G (ebioscience). A list of antibodies used can be found in **Supplementary Table 3**.

*Tissue and OCT-embedded organoids*

Tissue samples were fixed on ice for 30-45 min in 4% PFA. Tissues were then cryoprotected in 30% sucrose overnight on ice. Organoids were removed from matrigel using cell recovery solution and fixed for 20 min in 4% PFA and washed twice with PBS. Tissue and organoids were then embedded in OCT, snap frozen, and sectioned at 8 µm. Slides were blocked with 10% donkey serum/0.1% Triton-X for 1 h. Primary antibodies, diluted in 1% donkey serum/0.1% Triton-X (antibody diluent), were applied overnight at 4°C. Slides were washed three times for 5 min with antibody diluent. Secondary antibodies in antibody diluent were applied for 1h at RT. Slides were washed three times with antibody diluent, nuclei counter-stained with Hoechst 33258, and mounted with Fluoromount G. All images were acquired using either a Zeiss LSM700 laser scanning confocal or a Leica DMLB fluorescent microscope and analyzed using ImageJ.

**Flow cytometry**

Primary epithelium and/or organoids were dissociated to single cells using TrypLE Express. Cell staining and flow cytometry were performed as previously described (1).

**Quantitative RT-PCR (qPCR)**

Total RNA was extracted using either the RNeasy Mini or Micro Kit (Qiagen) depending on the expected RNA yield. Total RNA was reverse transcribed using Superscript II Reverse Transcriptase (Invitrogen). 10μL reactions were prepared using the SensiMix SYBR Low-ROX Kit (Bioline) and run on either a Life Technologies QuantStudio 12K Flex or QuantStudio 6 machine in technical duplicate. All genes were normalized to the housekeeping gene Ubiquitin (UBC). Primer sequences are listed in **Supplementary Table 4**.

**Primary human controls for qPCR**

*Human hepatocytes* were purchased from Biopredic International, France. *Human sigmoid colon organoids* were derived from biopsy samples and cultured as described previously (2). Intestinal biopsies for the generation of human sigmoid colon organoids were obtained following ethical approval and informed consent (REC-12/EE/0482, REC-17/EE/0265). *Human embryonic stem cells* were cultured as previously described and the line H9 used for all experiments (3).

**Single Cell Clonality Assays**

Extrahepatic and intrahepatic bile duct organoids (IHBD_NO CHIR) were removed from matrigel and dissociated to single cells. Organoids were incubated at 4°C in cell recovery solution for 30 minutes to remove cells from the matrigel. Organoids were then incubated for 5 minutes in accutase to dissociate the organoids to single cells. Cells were washed once and pelleted at 300 x g. Viable cells were counted with tryphan blue and 1,000 single cells were re-seeded per well in matrigel. Single cells were then cultured for 7 days. The number of organoids formed in each well were counted on day 7.

**RNA-Sequencing**

RNA was isolated from 3-7 biological replicates from organoids at Passage 5 or mechanically dissociated extrahepatic epithelial enriched tissues as described above. RNA samples were sequenced at the NIAID Genomics Technologies Section. Poly-A purified mRNA truSeq libraries were prepared using the Illumina NeoPrep system. Single-end 76bp reads were obtained using a NextSeq500. At least 20 million reads/sample evenly distributed across the lanes were obtained. If this number of reads was not reached, the samples were re-sequenced and sequencing replicates merged. Reads were trimmed to 70 bp and reads less than 40 bp were discarded. Transcript abundance was estimated using *Salmon* with default settings (4). *Tximport* was used to summarize transcript abundances to the gene level and *DeSeq2* was used for principal component and differential gene expression analyses in R (5,6). Only protein coding genes in the Ensembl BioMart GRCh38 database were included in analyses. A significance cut-off for differentially expressed genes was set as False Discovery Rate (FDR) less than 0.05 and a log_2_FoldChange with an absolute value greater than 1.

Heatmaps of the variance stabilized transformed counts were generated using Morpheus (<https://software.broadinstitute.org/morpheus>). Hierarchical clustering was performed using one minus Pearson correlation with average linkage. Gene ontology (GO) enrichment analyses were performed using GOrilla with a significance cut off for enriched GO terms set as a FDR less than 0.05 using the Benjamini and Hochberg method (7).

**References**

1. Sampaziotis F, Justin AW, Tysoe OC, Sawiak S, Godfrey EM, Upponi SS, et al. Reconstruction of the mouse extrahepatic biliary tree using primary human extrahepatic cholangiocyte organoids. Nat. Med. 2017;23:954–963.

2. Kraiczy J, Nayak KM, Howell KJ, Ross A, Forbester J, Salvestrini C, et al. DNA methylation defines regional identity of human intestinal epithelial organoids and undergoes dynamic changes during development. Gut. 2017;0:1–13.

3. Vallier L, Alexander M, Pedersen RA. Activin/Nodal and FGF pathways cooperate to maintain pluripotency of human embryonic stem cells. J. Cell Sci. 2005;118:4495–4509.

4. Patro R, Duggal G, Love MI, Irizarry RA, Kingsford C. Salmon provides fast and bias-aware quantification of transcript expression. Nat. Methods. 2017;14:417–419.

5. Soneson C, Love MI, Robinson MD. Differential analyses for RNA-seq: transcript-level estimates improve gene-level inferences. F1000Research. 2016;4:1521.

6. Love MI, Huber W, Anders S. Moderated estimation of fold change and dispersion for RNA-seq data with DESeq2. Genome Biol. 2014;15:550.

7. **Eden E, Navon R,** Steinfeld I, Lipson D, Yakhini Z. GOrilla: a tool for discovery and visualization of enriched GO terms in ranked gene lists. BMC Bioinformatics. 2009;10:48.

*Co-first authors are indicated in **bold***
